# Cyclic Bis-1,3-dialkylpyridiniums from the Sponge *Haliclona* sp.

**DOI:** 10.3390/md10092126

**Published:** 2012-09-24

**Authors:** Yoonyeong Lee, Kyung Hwa Jang, Ju-eun Jeon, Woo-Young Yang, Chung J. Sim, Ki-Bong Oh, Jongheon Shin

**Affiliations:** 1 Natural Products Research Institute, College of Pharmacy, Seoul National University, San 56-1, Sillim, Gwanak, Seoul 151-742, Korea; Email: zizel18@snu.ac.kr (Y.L.); khjang@ucsd.edu (K.H.J.); mirabn@snu.ac.kr (J.-e.J.); 2 Department of Agricultural Biotechnology, College of Agriculture & Life Science, Seoul National University, San 56-1, Sillim, Gwanak, Seoul 151-921, Korea; Email: yang0829@snu.ac.kr; 3 Department of Biological Science, College of Life Science and Nano Technology, Hannam University, 461-6 Jeonmin, Yuseong, Daejeon 305-811, Korea; Email: cjsim@hnu.kr

**Keywords:** *Haliclona* sp., bis-1,3-dialkylpyridiniums, cyclostellettamines, cytotoxicity, antimicrobial activity

## Abstract

Eight novel cyclic bis-1,3-dialkylpyridiniums, as well as two known compounds from the cyclostellettamine class, were isolated from the sponge *Haliclona* sp. from Korea. Structures of these novel compounds were determined using combined NMR and FAB-MS/MS analyses. Several of these compounds exhibited moderate cytotoxic and antibacterial activities against A549 cell-line and Gram-positive strains, respectively. The structure-activity relationships of cyclostellettamines are discussed based on their bioactivities.

## 1. Introduction

The 1,3-dialkylpyridiniums (with both cyclic and linear frameworks) are widely distributed cytotoxic compounds that play a role in the antimicrobial properties of marine sponges of the order Haplosclerida (genera *Amphimedon*, *Haliclona*, and *Xestospongia*) [1,2,3,4]. Previous studies have evaluated 1,3-dialkylpyridinium inhibition of histone deacetylase (HDAC) [5,6] and epidermal growth factor (EGF) receptor-mediated mitogenesis [7]. These compounds are also known to block the interaction of chemokines [8] and the binding of methyl quinuclidinyl benzilate (QNB) to the muscarinic receptor [9,10]. In addition to their chemical and bioactivity properties, these metabolites are considered to be the biogenetic precursors of a wide variety of compounds with complicated carbon skeletons and diverse functionalities, such as halicyclamines, ingenamines, manzamines, and sarains [2].

When evaluating bioactive metabolites from Korean marine invertebrates, we recently identified haliclonin A, a cytotoxic macrocylic bisamide from the sponge *Haliclona* sp. [11]. The novel carbon skeleton of this compound was highly reminiscent of bis-1,3-dialkylpyridiniums, especially cyclostellettamines isolated from *Haliclona* sp. [9,12]. Thus, we searched for similar 1,3-dialkylpyridinium metabolites in the polar fractions of extracts from diverse collections of this sponge. Here, we report eight novel derivatives of cyclostellettamines with diverse alkyl chains. Several of these compounds exhibited moderate cytotoxicity against the lung cancer A549 cell-line and antibacterial activities against Gram-positive strains. Additional information on their structure-activity relationships were deduced based on their cytotoxicity and antibacterial activity.

## 2. Results and Discussion

**Figure 1:**
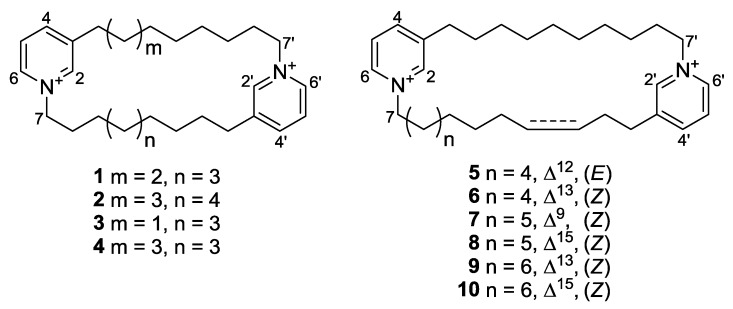
Structures of compounds **1**–**10**.

The sponge *Haliclona* sp. specimens were lyophilized, macerated, and repeatedly extracted with CH_2_Cl_2_ and MeOH. The combined extracts were separated by solvent-partitioning followed by ODS vacuum flash chromatography. The highly polar H_2_O-MeOH (50:50) chromatographic fraction was further fractionated by Sephadex LH-20 gel-permeation chromatography and separated by ODS-HPLC to yield 10 compounds (Figure 1). The structures of compounds **1** and **2** were identified as cyclostellettamine N and Q, respectively, based on spectroscopic analyses and a comparison of NMR and FAB-MS/MS data [13,14].

The molecular formula of compound **3**, with the shortest retention time in reversed-phase HPLC, was C_28_H_42_Cl_2_N_2_ based on the quasi-molecular ion clusters at *m/z* 407.3428 [M − H]^+^ and 443.3195 [M + Cl]^+^ in the HR-FAB-MS data. The spectroscopic data of this compound were very similar to those of **1** and **2** (Table 1 and Table 2). 

**Table 1:** ^13^C NMR (ppm, mult) assignments for compounds **3**–**10** in MeOH-*d*_4_.

Position	3		4		5		6		7		8		9		10	
2	145.5,	CH	145.6,	CH	145.3,	CH	145.3,	CH	145.2,	CH	145.6,	CH	145.2,	CH	145.3,	CH
3	145.3,	C	145.3,	C	145.7,	C	145.5,	C	146.7,	C	145.3,	C	145.7,	C	145.5,	C
4	147.0,	CH	146.9,	CH	146.9,	CH	147.0,	CH	146.9,	CH	146.9,	CH	146.9,	CH	147.0,	CH
5	129.2,	CH	129.2,	CH	129.2,	CH	128.8,	CH	129.2,	CH	129.1,	CH	129.1,	CH	129.2,	CH
6	143.4,	CH	143.4,	CH	143.4,	CH	143.5,	CH	143.5,	CH	143.4,	CH	143.5,	CH	143.4,	CH
7	62.7,	CH_2_	62.8,	CH_2_	62.8,	CH_2_	62.8,	CH_2_	62.9,	CH_2_	62.9,	CH_2_	62.9,	CH_2_	62.8,	CH_2_
8	32.2,	CH_2_	32.1,	CH_2_	32.2,	CH_2_	32.3,	CH_2_	24.8,	CH_2_	32.3,	CH_2_	32.3,	CH_2_	32.1,	CH_2_
9	26.7,	CH_2_	26.5,	CH_2_	26.2,	CH_2_	26.7,	CH_2_	137.6,	CH	26.9,	CH_2_	26.8,	CH_2_	26.8,	CH_2_
10	29.5 *^a^*,	CH_2_	29.6 *^b^*,	CH_2_	29.6,	CH_2_	30.0 *^d^*,	CH_2_	124.7,	CH	29.7 *^f^*,	CH_2_	30.5,	CH_2_	30.1 *^h^*,	CH_2_
11	29.8 *^a^*,	CH_2_	30.0 *^b^*,	CH_2_	32.7,	CH_2_	30.2,	CH_2_	28.1,	CH_2_	30.3 *^f^*,	CH_2_	30.7,	CH_2_	30.2 *^h^*,	CH_2_
12	29.9 *^a^*,	CH_2_	30.0 *^b^*,	CH_2_	132.3,	CH	28.0,	CH_2_	30.5,	CH_2_	30.1 *^f^*,	CH_2_	28.0,	CH_2_	30.2 *^h^*,	CH_2_
13	30.2 *^a^*,	CH_2_	30.1 *^b^*,	CH_2_	130.7,	CH	133.3,	CH	29.2 *^e^*,	CH_2_	30.9,	CH_2_	131.1,	CH	30.7,	CH_2_
14	29.2,	CH_2_	29.2,	CH_2_	32.4,	CH_2_	128.0,	CH	29.2 *^e^*,	CH_2_	28.3,	CH_2_	130.4,	CH	28.2,	CH_2_
15	31.3,	CH_2_	31.2,	CH_2_	31.1,	CH_2_	29.2,	CH_2_	29.6 *^e^*,	CH_2_	135.7,	CH	27.9,	CH_2_	133.5,	CH
16	33.2,	CH_2_	33.1,	CH_2_	33.2,	CH_2_	33.2,	CH_2_	31.1,	CH_2_	125.1,	CH	29.7,	CH_2_	127.8,	CH
17	-		-		-		-		33.1,	CH_2_	31.0,	CH_2_	31.3,	CH_2_	29.1,	CH_2_
18	-		-		-		-		-		-		33.3,	CH	33.2,	CH_2_
2′	145.5,	CH	145.6,	CH	145.3,	CH	145.5,	CH	145.2,	CH	144.6,	CH	145.2,	CH	145.5,	CH
3′	145.4,	C	145.3,	C	145.5,	C	144.9,	C	145.7,	C	144.7,	C	145.7,	C	145.0,	C
4′	146.9,	CH	146.9,	CH	146.9,	CH	147.2,	CH	147.0,	CH	146.7,	CH	146.9,	CH	147.3,	CH
5′	129.2,	CH	129.2,	CH	129.1,	CH	129.2,	CH	129.2,	CH	129.2,	CH	129.1,	CH	128.9,	CH
6′	143.5,	CH	143.4,	CH	143.5,	CH	143.5,	CH	143.5,	CH	143.7,	CH	143.5,	CH	143.4,	CH
7′	62.7,	CH_2_	62.8,	CH_2_	62.8,	CH_2_	62.9,	CH_2_	62.8,	CH_2_	62.8,	CH_2_	62.8,	CH_2_	62.9,	CH_2_
8′	31.9,	CH_2_	32.1,	CH_2_	32.2,	CH_2_	32.4,	CH_2_	32.0,	CH_2_	32.2,	CH_2_	32.3,	CH_2_	32.6,	CH_2_
9′	26.4,	CH_2_	26.5,	CH_2_	26.6,	CH_2_	26.8,	CH_2_	26.5,	CH_2_	26.6,	CH_2_	26.7,	CH_2_	27.0,	CH_2_
10′	30.3 *^a^*,	CH_2_	29.6 *^b^*,	CH_2_	29.9 *^c^*,	CH_2_	29.7 *^d^*,	CH_2_	29.7 *^e^*,	CH_2_	29.1 *^f^*,	CH_2_	29.6 *^g^*,	CH_2_	30.3 *^h^*,	CH_2_
11′	30.4 *^a^*,	CH_2_	30.0 *^b^*,	CH_2_	29.9 *^c^*,	CH_2_	29.9 *^d^*,	CH_2_	30.1 *^e^*,	CH_2_	29.9 *^f^*,	CH_2_	29.9 *^g^*,	CH_2_	30.4 *^h^*,	CH_2_
12′	29.0,	CH_2_	30.0 *^b^*,	CH_2_	30.0 *^c^*,	CH_2_	30.0 *^d^*,	CH_2_	30.1 *^e^*,	CH_2_	29.9 *^f^*,	CH_2_	30.0 *^g^*,	CH_2_	30.5 *^h^*,	CH_2_
13′	30.9,	CH_2_	30.1 *^b^*,	CH_2_	30.0 *^c^*,	CH_2_	30.4 *^d^*,	CH_2_	30.2 *^e^*,	CH_2_	30.0 *^f^*,	CH_2_	30.1 *^g^*,	CH_2_	30.7 *^h^*,	CH_2_
14′	33.0,	CH_2_	29.2,	CH_2_	29.2,	CH_2_	28.7,	CH_2_	29.1,	CH_2_	29.1,	CH_2_	29.4,	CH_2_	29.1,	CH_2_
15′	-		31.2,	CH_2_	31.2,	CH_2_	30.7,	CH_2_	30.9,	CH_2_	30.4,	CH_2_	31.3,	CH_2_	31.1,	CH_2_
16′	-		33.1,	CH_2_	33.0,	CH_2_	32.9,	CH_2_	33.2,	CH_2_	33.1,	CH_2_	33.3,	CH_2_	33.1,	CH_2_

*^a–h^*: Interchangeable signals.

**Table 2:** ^1^H MMR (*δ*, mult, *J* in Hz) assignments for compounds **3**–**6** in MeOH-*d*_4_.

Position	3	4	5	6
2	8.96 (1H, s)	8.98 (1H, s)	8.95 (1H, s)	8.99 (1H, s)
3	-	-	-	-
4	8.45 (1H, d, 7.6)	8.46 (1H, d, 8.0)	8.46 (1H, d, 8.2)	8.45 (1H, d, 8.2)
5	8.02 (1H, dd, 7.7, 6.2)	8.02 (1H, dd, 7.7, 6.4)	8.02 (1H, dd, 7.4, 6.1)	8.01 (1H, dd, 7.4, 6.6)
6	8.83 (1H, dd, 6.0)	8.85 (1H, dd, 6.0)	8.83 (1H, br s)	8.84 (1H, dd, 6.2)
7	4.64 (1H, t, 6.8)	4.65 (2H, t, 6.8)	4.65 (2H, t, 7.2)	4.64 (2H, t, 6.9)
8	2.00 (2H, quint, 7.3)	2.01 (2H, tt, 7.4, 6.8)	2.03 (2H, m)	2.00 (2H, m)
9	1.26 (2H, m)	1.25 (2H, m)	1.29 (2H, m)	1.23 (2H, m)
10	1.25 ~ 1.40 (2H, m)	1.20 ~ 1.36 (2H, m)	1.42 (2H, m)	1.26 (2H, m)
11	1.25 ~ 1.40 (2H, m)	1.20 ~ 1.36 (2H, m)	2.01 (2H, m)	1.24 (2H, m)
12	1.25 ~ 1.40 (2H, m)	1.20 ~ 1.36 (2H, m)	5.41 (1H, br t, 3.6)	1.74 (2H, m)
13	1.25 ~ 1.40 (2H, m)	1.20 ~ 1.36 (2H, m)	5.41 (1H, br t, 3.6)	5.38 (2H, m)
14	1.30 (2H, m)	1.28 (2H, m)	2.04 (2H, m)	5.38 (2H, m)
15	1.72 (2H, quint, 7.4)	1.74 (2H, tt, 7.3, 7.3)	1.78 (2H, m)	2.46 (2H, dd, 12.9, 6.5)
16	2.89 (2H, 7.3)	2.90 (2H, t, 7.3)	2.88 (2H, t, 7.6)	2.95 (2H, t, 6.9)
17	-	-	-	-
18	-	-	-	-
2′	8.96 (1H, s)	8.98 (1H, s)	8.98 (1H, s)	8.95 (1H, s)
3′	-	-	-	-
4′	8.44 (1H, d, 7.7)	8.46 (1H, d, 8.0)	8.45 (1H, d, 6.8)	8.45 (1H, d, 8.2)
5′	8.02 (1H, dd, 7.7, 6.2)	8.02 (1H, dd, 7.7, 6.4)	8.01 (1H, dd, 7.4, 6.1)	8.02 (1H, dd, 7.4, 6.6)
6′	8.82 (1H, dd, 6.0)	8.85 (1H, dd, 6.0)	8.84 (1H, br s)	8.85 (1H, dd, 6.2)
7′	4.64 (1H, t, 6.5)	4.65 (2H, t, 6.8)	4.63 (2H, t, 6.7)	4.63 (2H, t, 7.5)
8′	2.02 (2H, tt, 7.8, 6.9)	2.01 (2H, tt, 7.4, 6.8)	2.00 (2H, m)	1.98 (2H, m)
9′	1.20 (2H, m)	1.25 (2H, m)	1.28 (2H, m)	1.24 (2H, m)
10′	1.25 ~ 1.40 (2H, m)	1.20 ~ 1.36 (2H, m)	1.27 ~ 1.40 (2H, m)	1.23 ~ 1.35 (2H, m)
11′	1.25 ~ 1.40 (2H, m)	1.20 ~ 1.36 (2H, m)	1.27 ~ 1.40 (2H, m)	1.23 ~ 1.35 (2H, m)
12′	1.20 (2H, m)	1.20 ~ 1.36 (2H, m)	1.27 ~ 1.40 (2H, m)	1.23 ~ 1.35 (2H, m)
13′	1.76 (2H, tt, 7.4, 7.2)	1.20 ~ 1.36 (2H, m)	1.27 ~ 1.40 (2H, m)	1.23 ~ 1.35 (2H, m)
14′	2.91 (2H, t, 7.0)	1.28 (2H, m)	1.30 (2H, m)	1.25 (2H, m)
15′	-	1.74 (2H, tt, 7.3, 7.3)	1.73 (2H, m)	1.77 (2H, m)
16′	-	2.90 (2H, t, 7.3)	2.89 (2H, t, 7.0)	2.92 (2H, t, 6.9)

**Figure 2:**
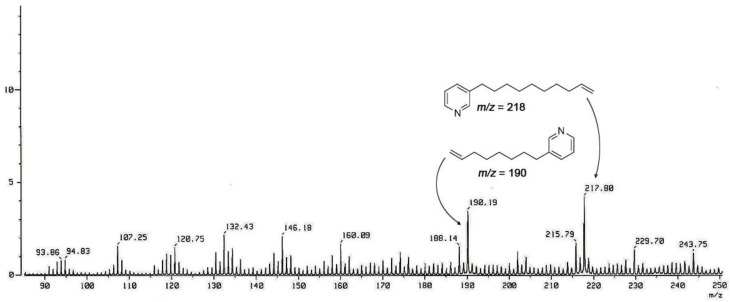
Positive FAB-MS/MS spectrum of compound **3**.

The UV absorption at 267 nm was reminiscent of two di-substituted pyridine rings, which was corroborated by aromatic proton signals [*δ*_H_, 8.96 (2 H, br s, H-2, H-2′), 8.45 (1 H, d, *J* = 7.6 Hz, H-4), 8.44 (1 H, d, *J* = 7.7 Hz, H-4′), 8.02 (2 H, dd, *J* = 7.7, 6.2 Hz, H-5, H-5′), 8.83 (1 H, dd, *J* = 6.0 Hz, H-6), 8.82 (1 H, dd, *J* = 6.0 Hz, H-6′)] and carbon signals [*δ*_C_ 145.5 (C-2, C-2′), *δ*_C_ 145.3 (C-3), *δ*_C_ 145.4 (C-3′), *δ*_C_ 147.0 (C-4), *δ*_C_ 146.9 (C-4′), *δ*_C_ 129.2 (C-5, C-5′), *δ*_C_ 143.4 (C-6), *δ*_C_ 143.5 (C-6′)] in the NMR data. Although the two and three linearly contiguous methylene units attached to the pyridinium (N-1, N-1′, C-3, and C-3′) were identified based on ^1^H COSY and *g*HMBC, only a large methylene envelope at *δ*_H_ 1.25–1.40 was observed in the ^1^H NMR spectrum, indicating that **3** was composed of two di-substituted pyridine rings and long aliphatic chains, forming a highly symmetric cyclic framework. The lengths of the methylene chains were determined based on the FAB-MS/MS data, which yielded two intense peaks at *m/z* 190 and 218, as shown in Figure 2. Since these daughter ions were attributable to a pair of monomeric products generated via Hoffmann-type elimination^7^ from their parent ion, **3** was structurally defined to be a cyclic hetero-dimer composed of C_8_ and C_10_ chains connecting the N-1 and C-3′ (also N-1′ and C-3) of pyridiniums.

The molecular formula of compound **4** was C_30_H_46_Cl_2_N_2_ based on HR-FAB-MS data with ion clusters at *m/z* 435.3741 [M − H]^+^ and 471.3503 [M + Cl]^+^. Thus, the structure consisted of two CH_2_ units larger than **3**. The NMR data of this compound were very similar to those of **3**, indicative of the same cycloalkylpyridinium nature. The FAB-MS/MS spectrum exhibited two intense daughter ions at *m/z* 218, confirming the symmetrical structure of **4**, which contained two C_10_ linear alkyl chains connecting the N-1 and C-3 between two pyridiniums.

The molecular formulas of compounds **5** and **6** were both C_30_H_44_Cl_2_N_2_ based on HR-FAB-MS analyses. The ^1^H NMR spectra of these compounds were almost identical and consisted of two 1,3-disubstituted pyridiniums, two methylene chains, and two olefinic protons [**5**: *δ*H 5.41 (2 H, br t, *J* = 3.6 Hz, H-12, H-13); **6**: *δ*H 5.38 (2 H, m, H-13, H-14)]. Two prominent daughter ions at *m/z* 216 and 218 in the FAB-MS/MS data of **5 **and **6 **showed a C_10_-saturated chain and a C_10_-chain with one double bond. The *g*HMBC data of **5** and **6** showed that these compounds had the same 1,3-dialkylpyridinium substitution patterns as **1**–**4**.

The remaining alkyl substructure and the locations of the double bonds were determined by combined NMR analyses. The ^1^H COSY (**5** and **6**: H-7-H-16) and *g*HMBC (**5**: H-11/C-12, C-13, H-12/C-11, C-14, H-13/C-11, C-14, H-14/C-12, C-13, **6**: H-12/C-13, C-14, H-13/C-12, C-15, H-14/C-12, C-15, H-15/C-13, C-14) data indicated that **5** and **6** contained a double bond at C-12 and C-13, respectively. Since the olefinic and allylic protons of the double bonds were chemically equivalent, the NOESY analysis could not be used to determine the configurations of the double bonds. Consequently, the configuration of the double bond in **5 **was assigned as *E* based on the downfield shifts of the allylic methylene [*δ*_C_ 32.7 (C-11) and 32.4 (C-14)], while the *Z*-configuration was deduced for **6** from the upfield chemical shifts of the allylic methylene [*δ*_C_ 28.0 (C-12) and 29.2 (C-15)] [15].

The molecular formulas of **7** and **8** were C_31_H_47_Cl_2_N_2_ based on HR-FAB-MS analyses. As found for **5** and **6**, the ^1^H NMR spectra of these compounds revealed characteristic features of cyclostellettamines: 1,3-disubstituted pyridines, methylene chains, and two olefinic protons [**7**: 5.37 (1 H, ddd, *J* = 11.4, 6.7, 2.0 Hz, H-9), 5.36 (1 H, ddd, *J* = 11.4, 6.7, 2.0 Hz, H-10); **8**: *δ*H 5.71 (1 H, dt, *J* = 10.8, 7.3 Hz, H-15), 5.66 (1 H, dt, *J* = 10.8, 7.3 Hz, H-16)] (Table 1 and Table 3). The FAB-MS/MS analyses of **7** and **8** supported the presence of a C_10_-saturated chain and a C_11_-chain with one double bond. The ^1^H COSY (**7**: H-7–H-12, H-16–H-17, **8**: H-7–H-9, H-13–H-17) and *g*HMBC (**7**: H-8/C-9, C-10, H-9/C-8, C-11, H-10/C-8, C-11, H-11/C-8, C-10, **8**: H-14/C-15, C-16, H-15/C-14, C-17, H-16/C-14, C-17, H-17/C-15, C-16) data placed the double bonds at C-9 and C-15 for **7** and **8**, respectively. The configurations of these double bonds were both determined to be *Z* based on the chemical shifts of the allylic methylene carbons [7: 24.8 (C-8) and 28.1 (C-11), **8**: *δ*C 28.3 (C-14) and 31.0 (C-17)] [15].

**Table 3:** ^1^H MMR (*δ*, mult, *J* in Hz) assignments for compounds **7**–**10** in MeOH-*d*_4_.

Position	7	8	9	10
2	8.99 (1H, s)	8.87 (1H, s)	8.99 (1H, s)	8.93 (1H, s)
3	-	-	-	-
4	8.43 (1H, d, 8.0)	8.46 (1H, d, 7.6)	8.45 (1H, d, 8.0)	8.45 (1H, d, 8.1)
5	8.00 (1H, dd, 7.8, 6.3)	8.03 (1H, dd, 7.8, 6.1)	8.02 (1H, dd, 7.7, 6.2)	8.02 (1H, dd, 7.9, 6.3)
6	8.80 (1H, dd, 6.2)	8.85 (1H, dd, 6.0)	8.82 (1H, dd, 6.1)	8.82 (1H, dd, 6.5)
7	4.62 (2H, t, 7.1)	4.65 (2H, t, 7.0)	4.61 (2H, t, 7.1)	4.64 (2H, t, 6.9)
8	2.10 (2H, m)	1.98 (2H, m)	2.00 (2H, m)	2.00 (2H, m)
9	5.37 (1H, ddd, 11.4, 6.7, 2.0)	1.26 (2H, m)	1.29 (2H, m)	1.24 (2H, m)
10	5.36 (1H, ddd, 11.4, 6.7, 2.0)	1.20 ~ 1.35 (2H, m)	1.32 (2H, m)	1.20 ~ 1.32 (2H, m)
11	1.94 (2H, m)	1.20 ~ 1.35 (2H, m)	1.28 (2H, m)	1.20 ~ 1.32 (2H, m)
12	1.36 (2H, m)	1.20 ~ 1.35 (2H, m)	1.99 (2H, m)	1.20 ~ 1.32 (2H, m)
13	1.23 ~ 1.35 (2H, m)	1.36 (2H, m)	5.34 (1H, m)	1.20 ~ 1.32 (2H, m)
14	1.23 ~ 1.35 (2H, m)	2.15 (2H, m)	5.32 (1H, m)	1.70 (2H, m)
15	1.23 ~ 1.35 (2H, m)	5 71 (1H, dt, 10.8, 7.3)	2.06 (2H, m)	5.39 (1H, dt, 10.6, 6.7)
16	1.78 (2H, m)	5.66 (1H, dt, 10.8, 7.3)	1.38 (2H, m)	5.38 (1H, dt, 10.6, 6.7)
17	2.88 (2H, t, 7.2)	3.68 (2H, t, 7.3)	1.72 (2H, m)	2.46 (2H, dt, 13.5, 6.6)
18	-	-	2.90 (2H, t, 7.5)	2.95 (2H, t, 6.8)
2′	8.90 (1H, s)	8.79 (1H, s)	8.98 (1H, s)	8.89 (1H, s)
3′	-	-	-	-
4′	8.45 (1H, d, 8.0)	8.47 (1H, d, 7.6)	8.45 (1H, d, 8.0)	8.43 (1H, d, 8.1)
5′	8.02 (1H, dd, 7.8, 6.3)	8.03 (1H, dd, 7.8, 6.1)	8.01 (1H, dd, 7.7, 6.2)	8.01 (1H, dd, 7.9, 6.3)
6′	8.82 (1H, dd, 6.2)	8.83 (1H, dd, 6.0)	8.83 (1H, dd, 5.8)	8.83 (1H, dd, 6.5)
7′	4.60 (2H, t, 6.7)	4.63 (2H, t, 7.5)	4.63 (2H, t, 6.8)	4.61 (2H, t, 6.9)
8′	1.99 (2H, m)	2.02 (2H, m)	2.01 (2H, m)	1.98 (2H, m)
9′	1.24 (2H, m)	1.28 (2H, m)	1.27 (2H, m)	1.25 (2H, m)
10′	1.23 ~ 1.38 (2H, m)	1.26 ~ 1.35 (2H, m)	1.26 ~ 1.36 (2H, m)	1.25 ~ 1.32 (2H, m)
11′	1.23 ~ 1.38 (2H, m)	1.26 ~ 1.35 (2H, m)	1.26 ~ 1.36 (2H, m)	1.25 ~ 1.32 (2H, m)
12′	1.23 ~ 1.38 (2H, m)	1.26 ~ 1.35 (2H, m)	1.26 ~ 1.36 (2H, m)	1.25 ~ 1.32 (2H, m)
13′	1.23 ~ 1.38 (2H, m)	1.26 ~ 1.35 (2H, m)	1.26 ~ 1.36 (2H, m)	1.25 ~ 1.32 (2H, m)
14′	1.32 (2H, m)	1.34 (2H, m)	1.30 (2H, m)	1.29 (2H, m)
15′	1.73 (2H, m)	1.74 (2H, m)	1.73 (2H, m)	1.75 (2H, m)
16′	2.88 (2H, t, 7.0)	2.90 (2H, t, 7.2)	2.89 (2H, t, 7.3)	2.91 (2H, t, 7.0)

The molecular formulas of **9** and **10** were both C_32_H_49_Cl_2_N_2_ based on HR-FAB-MS analyses. Similar to compounds **5**–**8**, the ^1^H NMR spectra of **9** and **10** revealed a 1,3-dialkyl pyridinium structure with two olefinic protons [**9**: *δ*H 5.34 (1 H, m, H-13), 5.32 (1 H, m, H-14); **10**: 5.39 (1 H, dt, *J* = 10.6, 6.7 Hz, H-15), 5.38 (1 H, dt, *J* = 10.6, 6.7 Hz, H-16)]. The FAB-MS/MS analyses of these compounds confirmed the presence of a C_10_-saturated chain and a C_12_-chain with one double bond. The COSY (**9**: H-7–H-18, **10**: H-7–H-9, H-14–H-18) and *g*HMBC (**9**: H-12/C-13, C-14, H-13/C-12, C-15, H-13/C-12, C-15, H-15/C-13, C-14, **10**: H-14/C-15, C-16, H-15/C-14, C-17, H-16/C-14, C-17, H-17/C-15, C-16) data were indicative of a double bond at C-13 and C-15 for **9** and **10**, respectively. The configurations of these double bonds were both assigned as *Z* based on the upfield shifts of the allylic methylene carbons [**9**: *δ*C 28.0 (C-12), 27.9 (C-15), **10**: 28.2 (C-14), 29.1 (C-17)].

A comparison of the ^13^C and ^1^H NMR data revealed almost identical chemical shifts for both carbons and protons among all cyclic 1,3-dialkylpyridinium dimers **1**–**10** (Table 1–Table 3). These compounds were structurally similar to cyclostellettamines reported previously with differences in the length of the alkyl chains, as well as the presence of double bonds [9]. Compounds **5**–**10** were dehydrocyclostellettamines containing one double bond in the alkyl chains. Subsequently, the carbon chemical shifts of allylic methylenes and coupling constants between the olefinic protons were used to assign *E* or Z configurations to these double bonds.

The 1,3-dialkylpyridinium metabolites possess diverse cytotoxic and antimicrobial activities [16,17]. Thus, we evaluated the cytotoxic and antimicrobial activities of compounds **1**–**10**. Since **7** was isolated in a TFA salt form, we evaluated the influence of the counter ion on bioactivity. Thus, the TFA salt of **4** was prepared using reversed phase HPLC (H_2_O-MeOH, 80:20, 0.1% TFA) on **4**. Compounds **1**–**10** and **4a** exhibited moderate cytotoxicity against the A549 lung cancer cell line, comparable to doxorubicin (Table 4). On the other hand, they also displayed a diverse range of antibacterial activities against Gram-positive strains (compounds **2**, **4**, **8**, **9**, and **10** were the most potent). These results are partly consistent with the previous report that the bioactivities of cyclostellettamines were significantly influenced by the length of the alkyl chains [16]. In addition, as shown for **6** and **9**, the bioactivities were influenced by the existence and location of the double bond, although the alkyl chains were the same length. This suggests a mechanism-of-action based on the distances between the charged moieties and electron-rich sites, possibly through an interaction with ionic membrane sites. We also observed that the antibacterial activities of these compounds were generally weaker against Gram-negative than Gram-positive strains. Also, the influence of pyridinium counterions was negligible since there was no significant difference between the bioactivities of **4** and **4a**. Overall, these results increase our understanding of the structure-activity relationships of cyclostellettamines.

In addition to the antimicrobial and cytotoxic activity assays, compounds **1**–**10** were also tested against diverse pathogenic fungal strains and microbial enzymes, such as isocitrate lyase, sortase A, and Na^+^/K^+^-ATPase, and failed to display any bioactivity (LC_50_ > 100 μg/mL for fungi, ED_50_ > 100 μM for enzymes).

**Table 4:** Results of Bioactivity Tests.

Compound	MIC (μg/mL)						LC_50_ (μM) *^a^*
	Gram (+) Bacterium			Gram (−) Bacterium			A549
	A	B	C	D	E	F	
**1**	50	100	50	100	100	>100	22.1
**2**	12.5	25	3.125	25	25	>100	15.3
**3**	50	50	100	50	25	>100	24.0
**4**	50	100	6.25	100	100	>100	26.3
**5**	25	100	25	50	50	>100	25.9
**6**	50	100	50	100	100	>100	28.9
**7**	50	100	100	100	50	>100	89.4
**8**	12.5	25	3.125	50	50	>100	15.7
**9**	12.5	25	3.125	25	25	>100	14.7
**10**	12.5	25	3.125	25	50	>100	14.8
**4a**	25	100	50	100	100	>100	21.9
Ampicillin	0.39	0.78	0.39	0.78	0.39	6.25	
Doxorubicin							3.37

A: *Staphylococcus aureus* (ATCC 6538p); B: *Bacillus subtilis* (ATCC 6633); C: *Micrococcus luteus* (IFO 12708); D: *Salmonella typhimurium* (ATCC 14028); E: *Proteus vulgaris* (ATCC 3851); F: *Escherichia coli* (ATCC 35270); *^a^* The results of 48 h treatment were chosen.

## 3. Experimental Section

### 3.1. General Experimental Procedures

Optical rotation was measured on a JASCO digital polarimeter using a 1 cm cell. IR spectra were recorded on a JASCO FT/IR 4200 spectrophotometer. UV spectra were recorded on a Hitachi U-3010 spectrophotometer. Proton and carbon NMR spectra were measured at 600 and 150 MHz (**3**) on Bruker Avance 600 and 500 and 125 MHz (**1**, **2** and **4**–**10**) on Bruker Avance 500, respectively. Mass spectrometric data were obtained from the Korea Basic Science Research Institute (Daegu) on a JEOL JMS-700 mass spectrometer. All solvents used were spectral grade or distilled from glass prior to use.

### 3.2. Collection and Taxonomic Identification

*Haliclona* sp. specimens were collected by hand using SCUBA equipment (20 m depth) at Sagyeri off the shore of Jeju Island, Korea, in November 1999. The pale-purple sponge measured 65 × 55 mm with a thickness of 27 mm and produced slime strands. The surface had many mamillate forms of a hard but fragile consistency. The size of the oxea spicule was (170–198) × (8–10) μm. A voucher specimen (registry No. Spo. 63) was deposited at the Natural History Museum, Hannam University, Korea, under the curatorship of C.J.S. Collections of the same specimens were made at the nearby Chuja-do in November 2003, and at Sagyeri in September 2009 and June 2010. Since the morphological features and ^1^H NMR spectra of crude extracts from these collections were identical to those of the 1999 collection, chemical investigation was performed on the combined specimens.

### 3.3. Extraction and Isolation

The freshly collected specimens were frozen immediately and kept at −25 °C until use. The specimens (dried wt. 1.2 kg) were lyophilized, macerated, and extracted repeatedly with MeOH (3 L × 3) and CH_2_Cl_2_ (3 L × 2). The combined organic extract (940.3 g) was partitioned between *n*-BuOH and H_2_O, and the former (158.18 g) was repartitioned between 15% aqueous MeOH (24.43 g) and *n*-hexane (130.25 g). The aqueous MeOH layer was subjected to C_18_ reversed-phase vacuum flash chromatography using sequential mixtures of MeOH and H_2_O as eluents (six fractions in gradient, H_2_O–MeOH, from 50:50 to 0:100), and finally acetone. The H_2_O–MeOH (50:50) fraction (1.64 g) was gel-filtered on a Sephadex LH-20 column with MeOH. The bioactive fractions were separated by C_18_ reversed-phase semi-preparative HPLC (YMC ODS-A column, 1 × 25 cm, H_2_O–MeOH, 80:20) to yield, in order of elution, compounds **3**, **4**, **1**, **7**, **8**, **2**, **6**, **5**, **10**, and **9**. Final purification of these metabolites was then accomplished by reversed-phase HPLC (YMC-Pack CN column, 1 × 25 cm, H_2_O–MeOH (85:15) for **1**–**4**, H_2_O–MeOH (90:10) for **5**, **6**, and **8**–**10**, and gradient solvents of H_2_O–MeOH (85:15) to (40:60) and 0.1% TFA for 30 min for **7**). The overall yields were 14.9, 19.8, 7.5, 187.4, 11.3, 22.5, 6.0, 72.4, 12.6, and 10.5 mg for **1**–**10**, respectively.

Compound **3**: Yellow, amorphous solid; UV (MeOH) λ_max_ 267 (log *ε *= 3.7), IR (ZnSe) ν_max_ 2927, 1506, 1461 cm^−1^; ^1^H and ^13^C NMR data, see Table 1 and Table 2, respectively; HR-FAB-MS *m/z* 407.3428 [M − H]^+^ (calculated for C_28_H_43_N_2_, 407.3426) and 443.3195 [M + Cl]^+^ (calculated for C_28_H_44_N_2_Cl, 443.3193); FAB-MS/MS fragments: *m/z* 190.19 [M − C_15_H_24_N]^+^ and 217.80 [M − C_13_H_20_N]^+^.

Compound **4**: White, amorphous solid; UV (MeOH) λ_max_ 266 (log *ε *= 3.9), IR (ZnSe) ν_max_ 2924, 1505, 1467 cm^–1^; ^1^H and ^13^C NMR data, see Table 1 and Table 2, respectively; HR-FAB-MS *m/z* 435.3741 [M − H]^+^ (calculated for C_30_H_47_N_2_, 435.3739) and 471.3503 [M + Cl]^+^ (calculated for C_30_H_48_N_2_Cl, 471.3506); FAB-MS/MS fragments: *m/z* 218.05 [M − C_15_H_24_N]^+^ and 218.05 [M − C_15_H_24_N]^+^.

Compound **5**: Yellow, amorphous solid; UV (MeOH) λ_max_ 267 (log *ε *= 3.8), IR (ZnSe) ν_max_ 2926, 1505, 1459 cm^−1^; ^1^H and ^13^C NMR data, see Table 1 and Table 2, respectively; HR-FAB-MS *m/z* 433.3586 [M − H]^+^ (calculated for C_30_H_45_N_2_, 433.3583), 469.3351 [M + Cl]^+^ (calculated for C_30_H_46_N_2_Cl, 469.3350); FAB-MS/MS fragments: *m/z* 216.06 [M − C_15_H_24_N]^+^, 218.04 [M − C_15_H_22_N]^+^.

Compound **6**: Yellow, amorphous solid; UV (MeOH) λ_max_ 266 (log *ε *= 3.8), IR (ZnSe) ν_max_ 2927, 1505, 1458 cm^−1^; ^1^H and ^13^C NMR data, see Table 1 and Table 2, respectively; HR-FAB-MS *m/z* 433.3584 [M − H]^+^ (calculated for C_30_H_45_N_2_, 433.3583) and 469.3352 [M + Cl]^+^ (calculated for C_30_H_46_N_2_Cl, 469.3350); FAB-MS/MS fragments: *m/z* 216.07 [M − C_15_H_24_N]^+^ and 218.04 [M − C_15_H_22_N]^+^.

Compound **7**: Yellow, amorphous solid; UV (MeOH) λ_max_ 267 (log *ε *= 3.9), IR (ZnSe) ν_max_ 2927, 1631, 1507, 1460, 1203, 1129 cm^−1^; ^1^H and ^13^C NMR data, see Table 1 and Table 3 respectively; HR-FAB-MS *m/z* 447.3736 [M − H]^+^ (calculated for C_31_H_47_N_2_, 447.3739) and 561.7414 [M + CF_3_COO]^+^ (calculated for C_30_H_48_N_2_CF_3_COO, 561.7416); FAB-MS/MS fragments: *m/z* 217.92 [M − C_16_H_24_N]^+^ and 229.76 [M − C_15_H_24_N]^+^.

Compound **8**: Yellow, amorphous solid; UV (MeOH) λ_max_ 267 (log *ε *= 3.8), IR (ZnSe) ν_max_ 2924, 1506, 1467 cm^−1^; ^1^H and ^13^C NMR data, see Table 1 and Table 3, respectively; HR-FAB-MS *m/z* 447.3742 [M − H]^+^ (calculated for C_31_H_47_N_2_, 447.3739) and 483.3504 [M + Cl]^+^ (calculated for C_31_H_48_N_2_Cl, 483.3506); FAB-MS/MS fragments: *m/z* 218.11 [M − C_16_H_24_N]^+^ and 229.87 [M − C_15_H_24_N]^+^.

Compound **9**: Yellow, amorphous solid; UV (MeOH) λ_max_ 266 (log *ε *= 3.7), IR (ZnSe) ν_max_ 2925, 1505, 1459 cm^−1^; ^1^H and ^13^C NMR data, see Table 1 and Table 3, respectively; HR-FAB-MS *m/z* 461.3898 [M − H]^+^ (calculated for C_32_H_49_N_2_, 461.3896) and 497.3665 [M + Cl]^+^ (calculated for C_32_H_50_N_2_Cl, 497.3663); FAB-MS/MS fragments: *m/z* 218.19 [M − C_17_H_26_N]^+^ and 243.75 [M − C_15_H_24_N]^+^.

Compound **10**: Yellow, amorphous solid; UV (MeOH) λ_max_ 266 (log *ε *= 3.9), IR (ZnSe) ν_max_ 2925, 1505, 1458 cm^−1^; ^1^H and ^13^C NMR data, see Table 1 and Table 3, respectively; HR-FAB-MS *m/z* 461.3899 [M − H]^+^ (calculated for C_32_H_49_N_2_, 461.3896) and 497.3665 [M + Cl]^+^ (calculated for C_32_H_50_N_2_Cl, 497.3663); FAB-MS/MS fragments: *m/z* 218.19 [M − C_17_H_26_N]^+^ and 243.75 [M − C_15_H_24_N]^+^.

### 3.4. Preparation of TFA Salt of Compound ***4** (**4a**)*

A small amount of compound **4** (6.0 mg) was injected into the HPLC (YMC-Pack CN column, 1 × 25 cm, H_2_O–MeOH, 80:20, 0.1% TFA) to yield **4a** (4.8 mg): UV (MeOH) λ_max_ 266 (log *ε *= 3.8), IR (ZnSe) ν_max_ 2928, 1631, 1506, 1464, 1203, 1129 cm^–1^; HR-FAB-MS *m/z* 435.3741 [M − H]^+^ (calculated for C_30_H_47_N_2_, 435.3739) and 549.7306 [M + CF_3_COO]^+^ (calculated for C_30_H_48_N_2_CF_3_COO, 549.7309); FAB-MS/MS fragments: *m/z* 218.05 [M − C_15_H_24_N]^+^ and 218.05 [M − C_15_H_24_N]^+^.

### 3.5. Biological Assays

Cytotoxicity assays, antimicrobial assays, and isocitrate lyase, sortase A, and Na^+^/K^+^-ATPase inhibition assays were performed as described previously [18,19,20,21,22]. For the cytotoxicity test, an MTT viability assay was performed as previously described with slight modifications [18]. MTT was first prepared as a stock solution of 5 mg/mL in phosphate buffered saline (PBS, pH 7.2) and was filtered. At the end of the treatment period (24 h, 48 h, and 72 h), with three different test drug concentrations in triplicate, MTT solution (20 μL) was added to each well and then incubated for 4 h at 37 °C; then solubilizing buffer (10% sodium dodecyl sulfate dissolved in 0.01 N HCl, 100 μL) was added to each well. After overnight incubation, the 96-well plate was read by an enzyme-linked immunosorbent assay (ELISA) reader at 570 nm for absorbance to determine the cell (A549 cell line) viability. The viable cells produced a dark blue formazan product, whereas no such staining was formed by dead cells [19]. The LC_50_ value was defined as the concentration that resulted in a 50% decrease in cell viability compared to that of control reactions in the absence of an inhibitor. The values (mean ± SD) were calculated from the dose–response curves of three concentrations of each test sample in three independent experiments (*n* = 3). 

## 4. Conclusion

Eight novel cyclic bis-1,3-dialkylpyridiniums (**3**–**10**), as well as two known compounds (**1** and **2**) belong to the cyclostellettamine class, were isolated from the sponge *Haliclona* sp. from Korea. These compounds were structurally different from the previous reported cyclostelletamines in the lengths of the alkyl chains, as well as the presence of double bonds in the chains. Several of these new compounds exhibited moderate cytotoxicity against A549 cell-line and inhibitory activities against Gram-positive bacterial strains. As shown in **6** and **9**, the bioactivities were influenced by the existence and the location of a double bond. Overall, these results provide us better understanding of the structure-activity relationships of the cyclostellettamine class compounds.

## Supplementary Files

Supplementary File 1: PDF-Document (PDF, 746 KB)
