# Supplementary material for: Cyclic Bis-1,3-dialkylpyridiniums from the Sponge Haliclona sp
Source: Mar Drugs. 2012 Sep 24;10(9):2126–37. doi: 10.3390/md10092126 (PMC3475278; doi:10.3390/md10092126)
Supplement: Supplementary File 1: — PDF-Document (PDF, 746 KB) [file marinedrugs-10-02126-s001.pdf]

Supplementary Information

Figure S1. The ^{1}H NMR (500 MHz, MeOH-d_4) spectrum of Compound **3**.
Figure S2. The ^{13}C NMR (150 MHz, MeOH-d_4) spectrum of Compound **3**.
Figure S3. The 1H NMR (500 MHz, MeOH-d_4) spectrum of Compound **4**.
Figure S4. The 13C NMR (125 MHz, MeOH-d_4) spectrum of Compound **4**.
Figure S5. The 1H NMR (500 MHz, MeOH-d_4) spectrum of Compound **5**.
Figure S6. The 13C NMR (125 MHz, MeOH-d_4) spectrum of Compound **5**.
Figure S7. The 1H NMR (500 MHz, MeOH-d_4) spectrum of Compound **6**.
Figure S8. The 13C NMR (125 MHz, MeOH-d_4) spectrum of Compound **6**.
Figure S9. The 1H NMR (500 MHz, MeOH-d_4) spectrum of Compound **7**.
Figure S10. The 13C NMR (125 MHz, MeOH-d_4) spectrum of Compound **7**.
Figure S11. The 1H NMR (500 MHz, MeOH-d_4) spectrum of Compound **8**.
Figure S12. The 13C NMR (125 MHz, MeOH-d_4) spectrum of Compound **8**.
Figure S13. The 1H NMR (500 MHz, MeOH-d_4) spectrum of Compound **9**.
Figure S14. The 13C NMR (125 MHz, MeOH-d_4) spectrum of Compound **9**.
Figure S15. The 1H NMR (500 MHz, MeOH-d_4) spectrum of Compound **10**.
Figure S16. The 13C NMR (125 MHz, MeOH-d_4) spectrum of Compound **10**.
Figure S17. The cytotoxicity test results.

Figure S1. The ^{1}H NMR (600 MHz, MeOH-d_4) spectrum of Compound **3**.

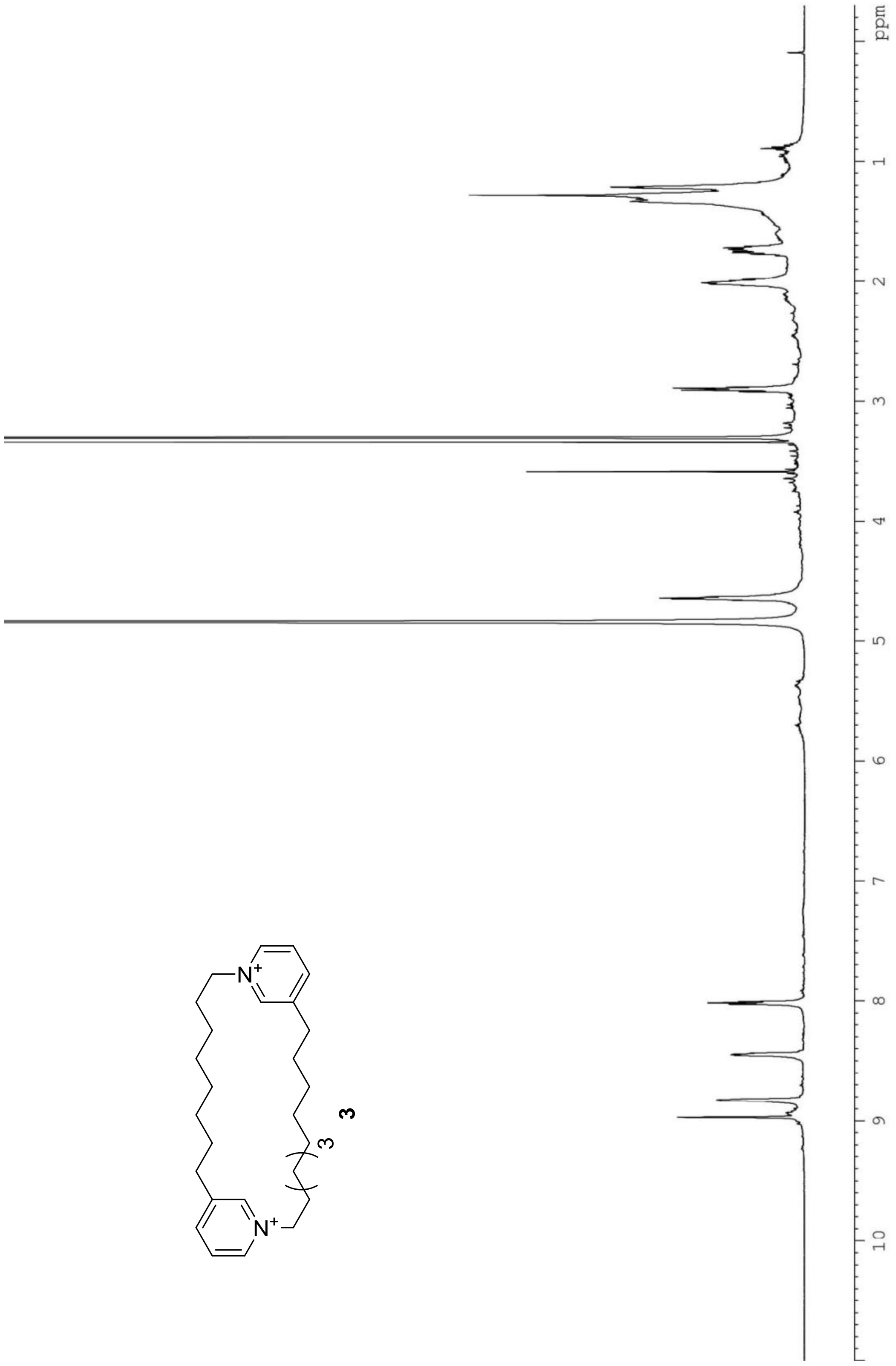

Figure S2. The 13C NMR (150 MHz, MeOH-d_4) spectrum of Compound **3**.

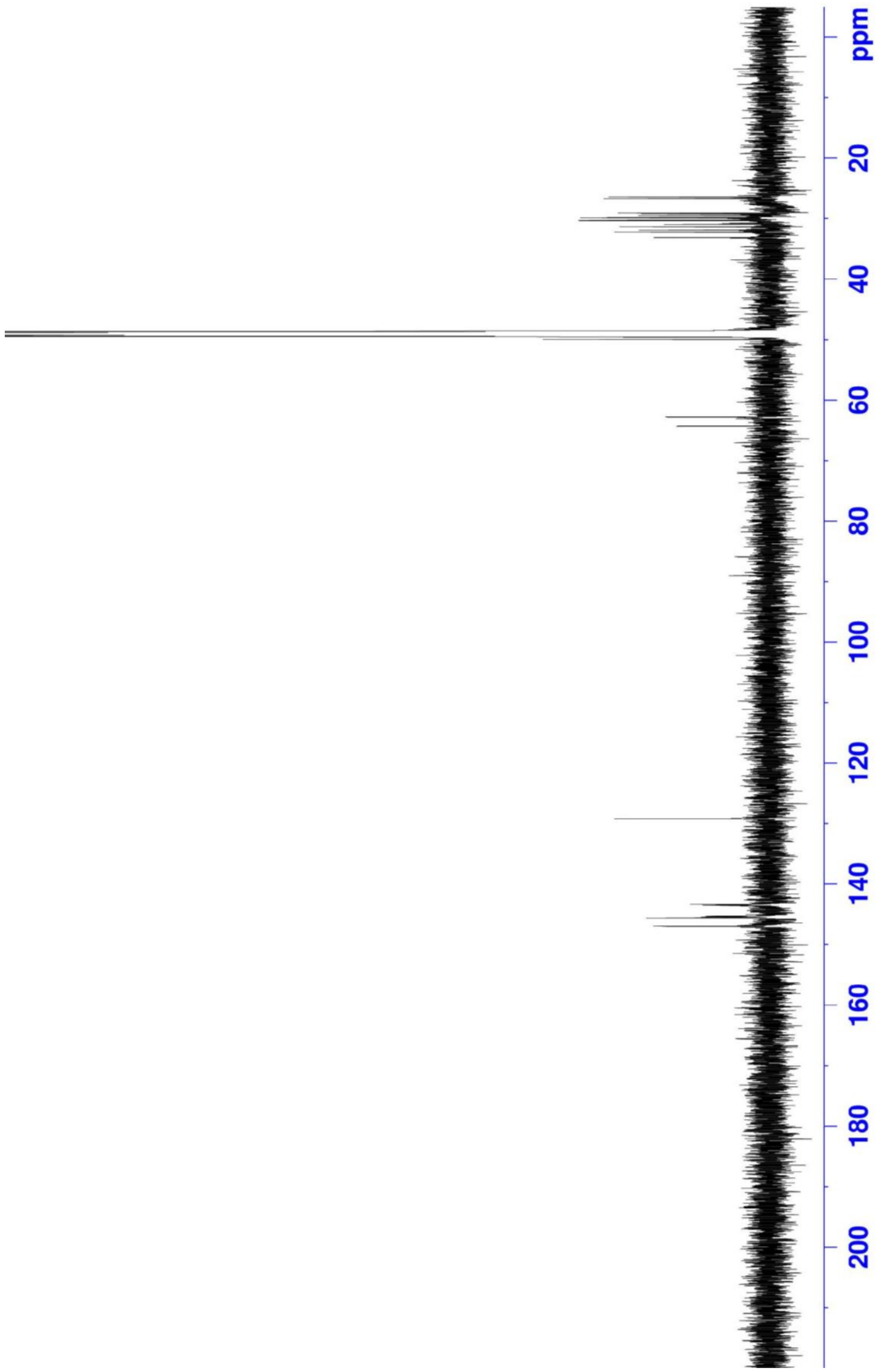

Figure S3. The 1H NMR (500 MHz, MeOH-d_4) spectrum of Compound **4**.

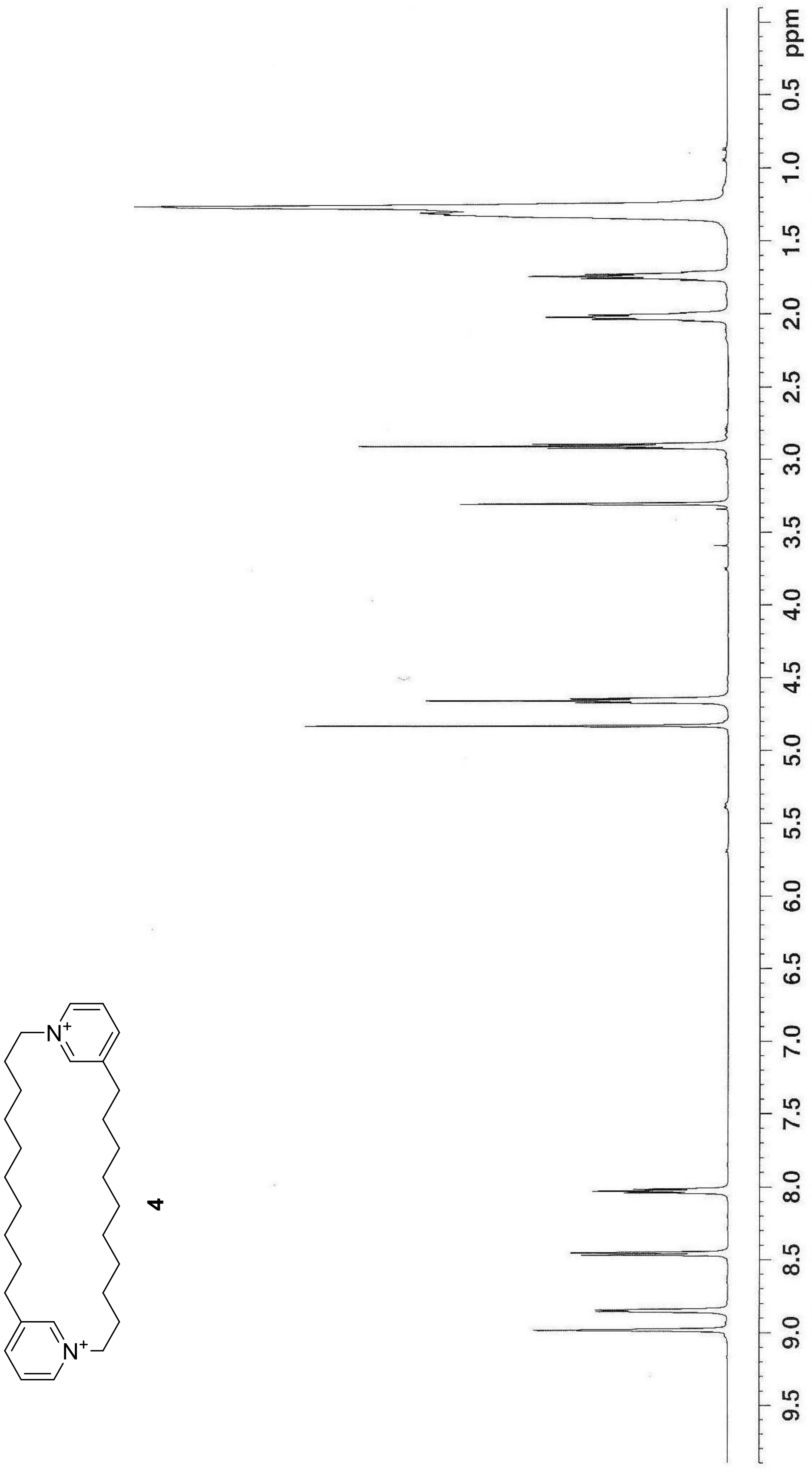

Figure S4. The 13C NMR (125 MHz, MeOH-d_4) spectrum of Compound **4**.

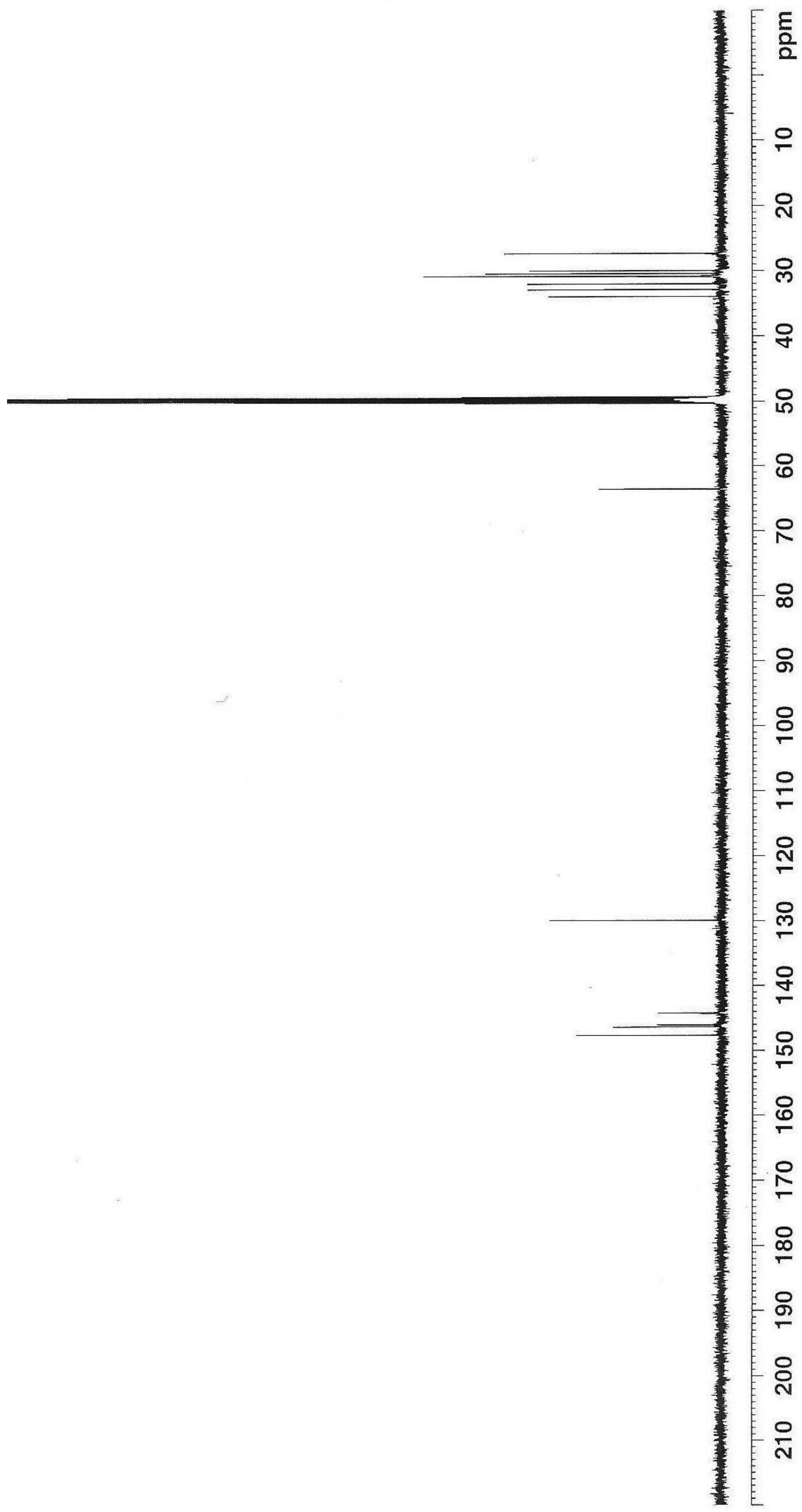

Figure S5. The 1H NMR (500 MHz, MeOH-d_4) spectrum of Compound **5**.

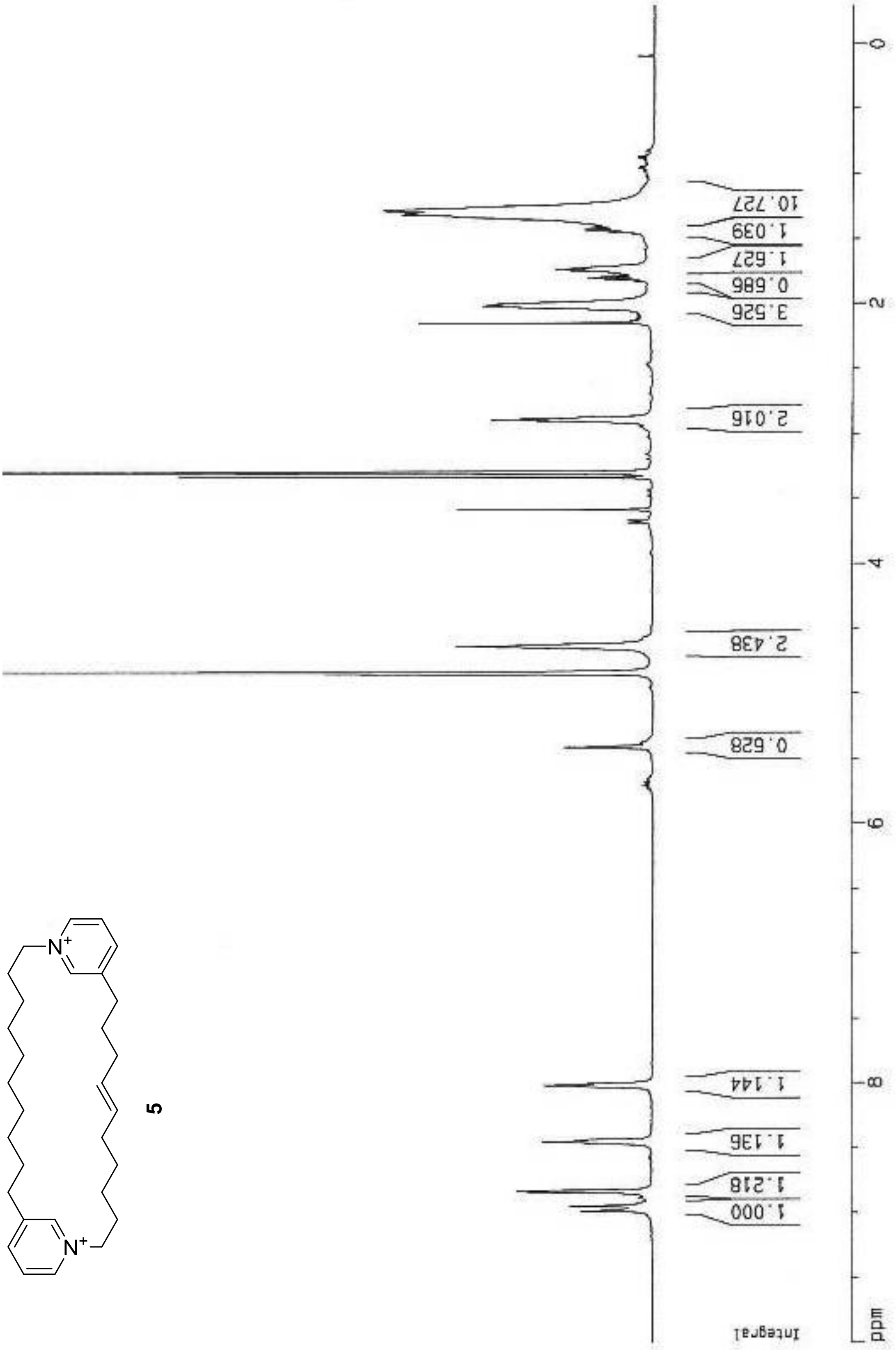

Figure S6. The 13C NMR (125 MHz, MeOH-d_4) spectrum of Compound **5**.

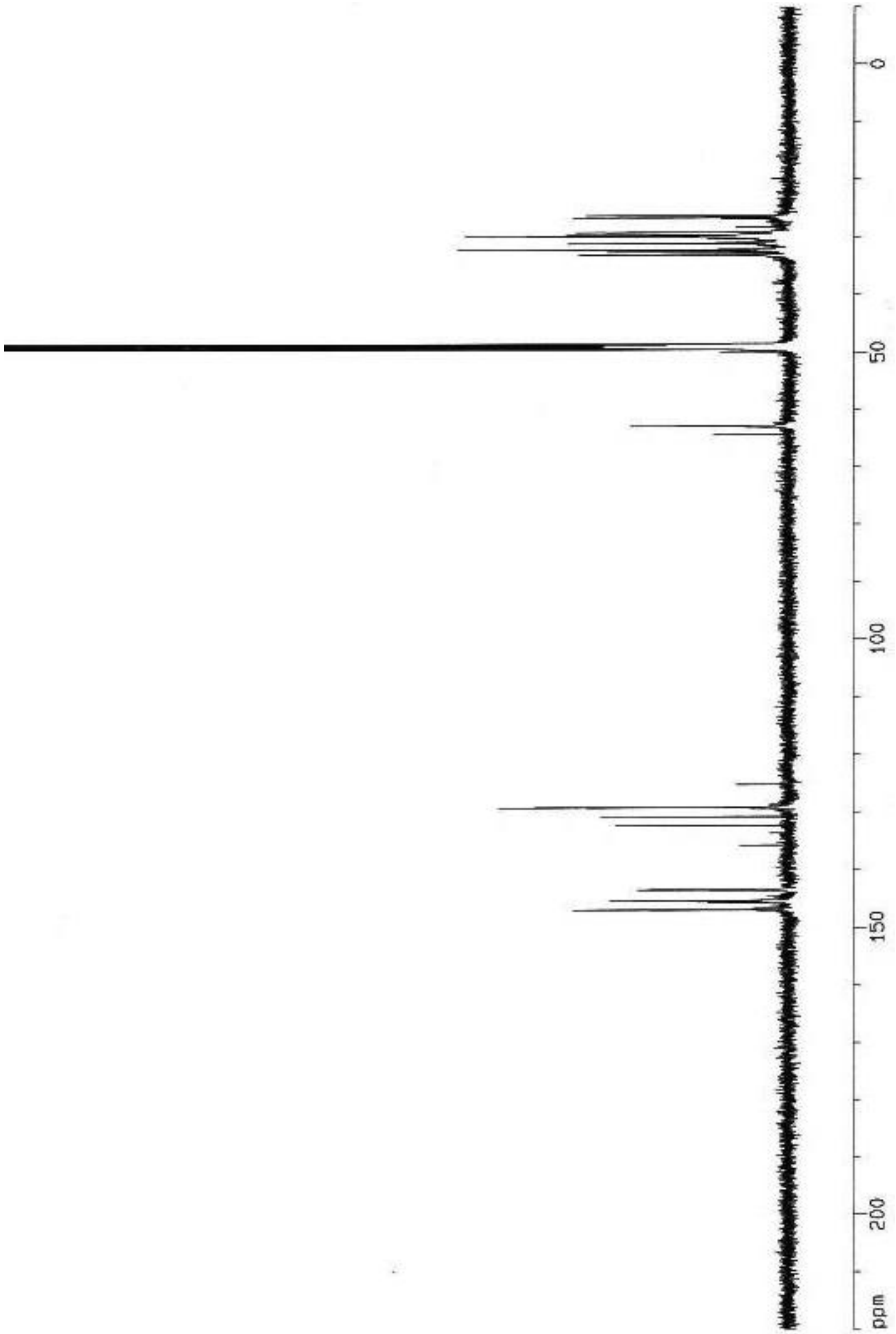

Figure S7. The 1H NMR (500 MHz, MeOH-d_4) spectrum of Compound **6**.

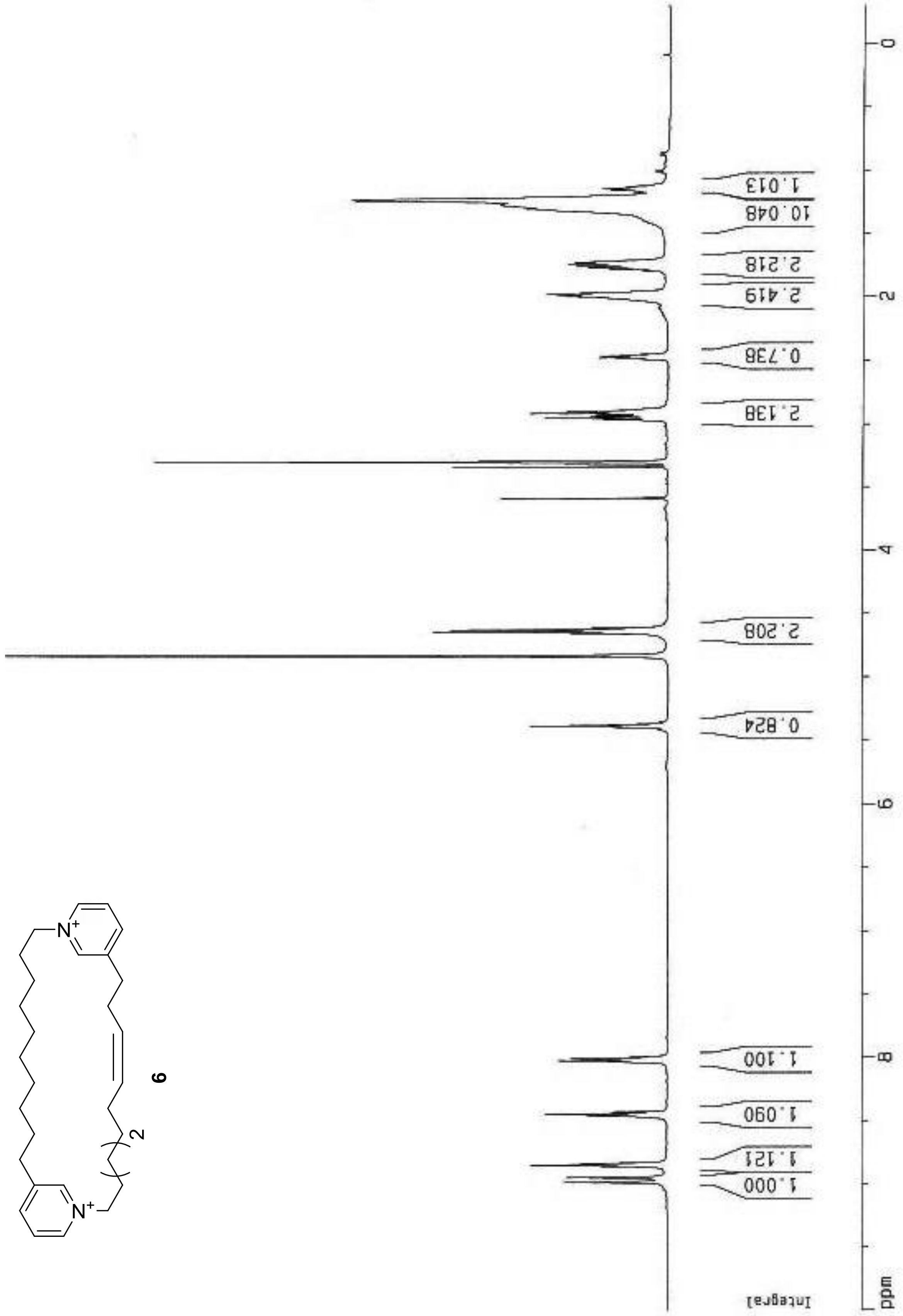

Figure S8. The 13C NMR (125 MHz, MeOH-d_4) spectrum of Compound **6**.

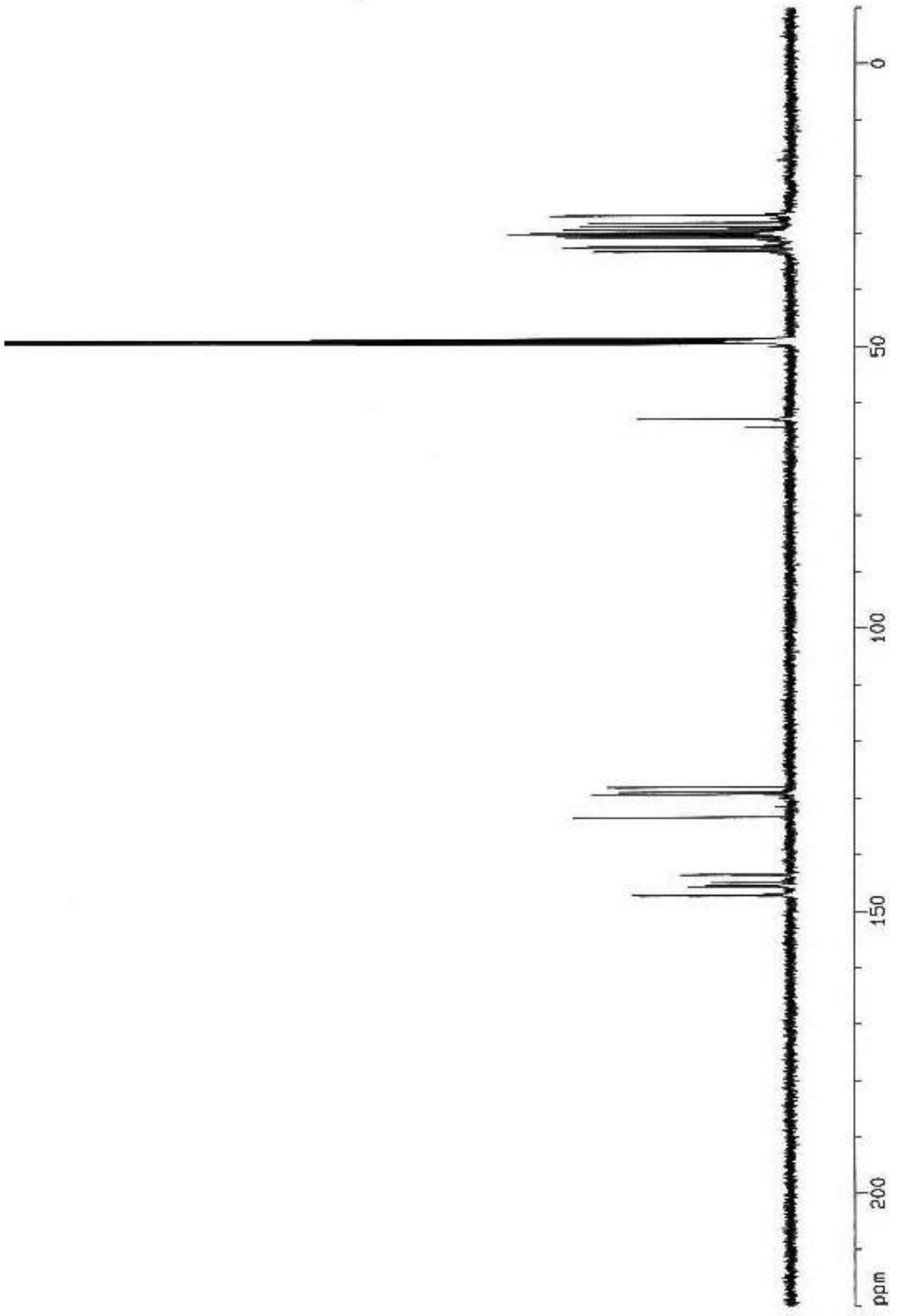

Figure S9. The 1H NMR (500 MHz, MeOH-d_4) spectrum of Compound **7**.

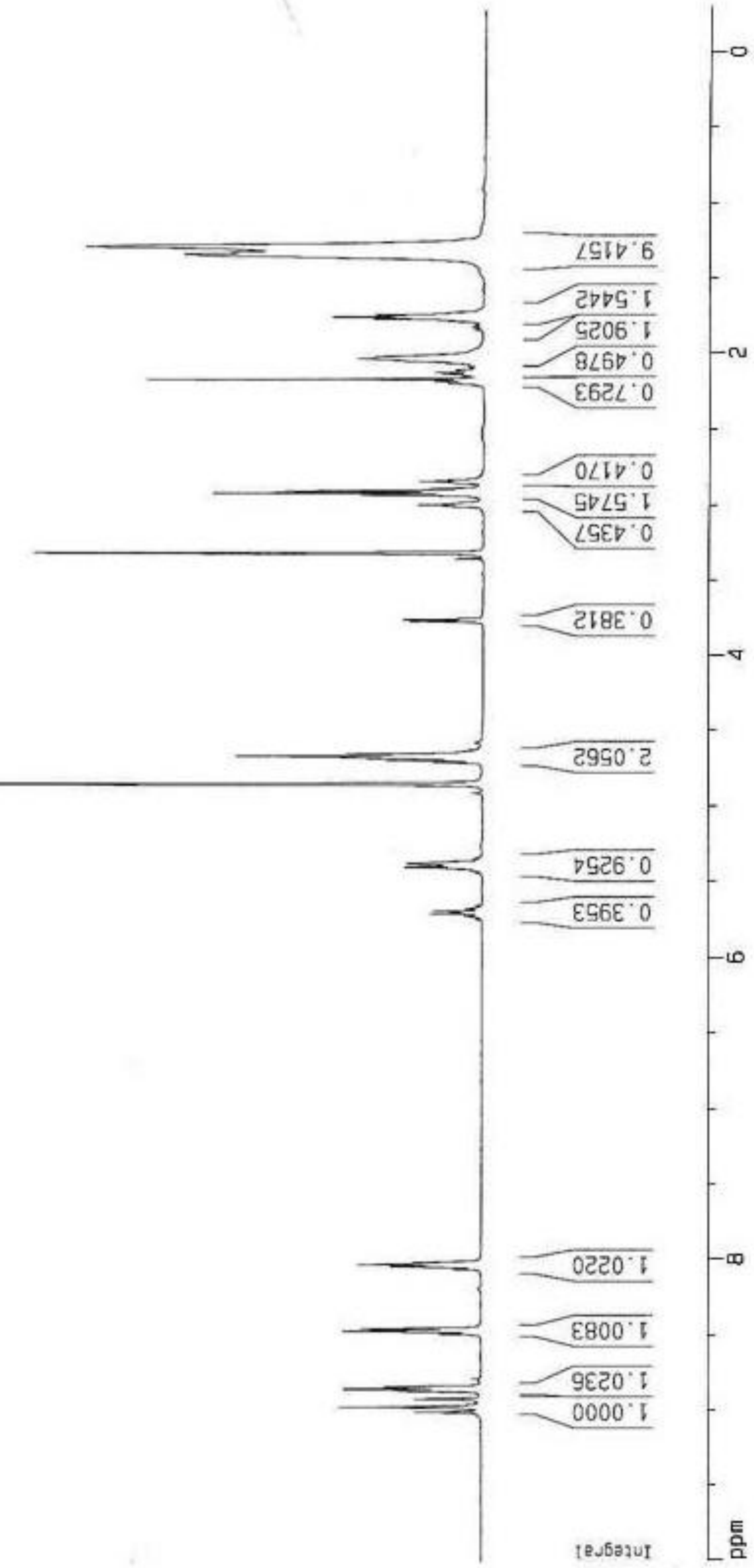

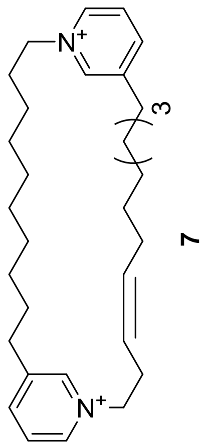

7

Figure S10. The 13C NMR (125 MHz, MeOH-d_4) spectrum of Compound **7**.

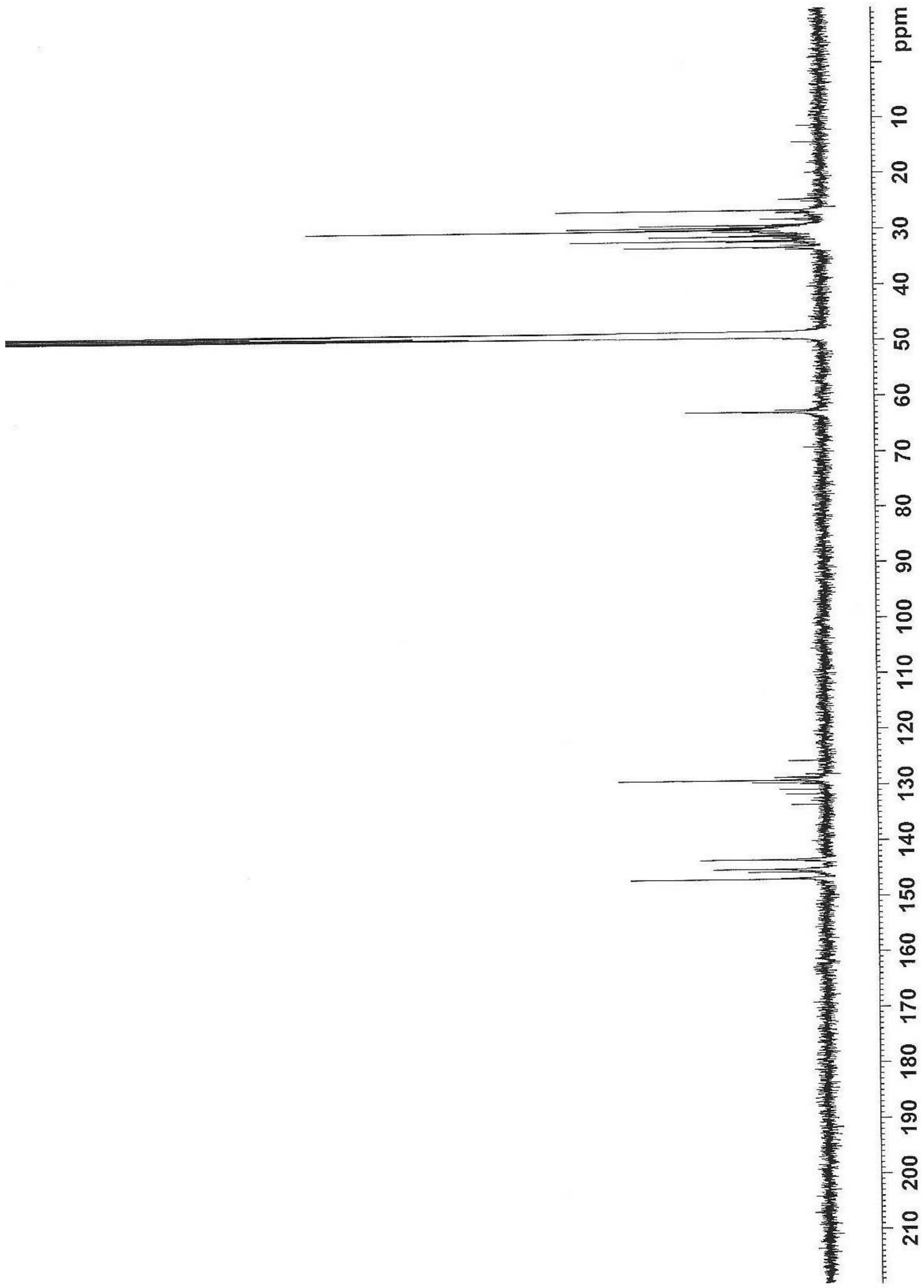

Figure S11. The 1H NMR (500 MHz, MeOH-d_4) spectrum of Compound **8**.

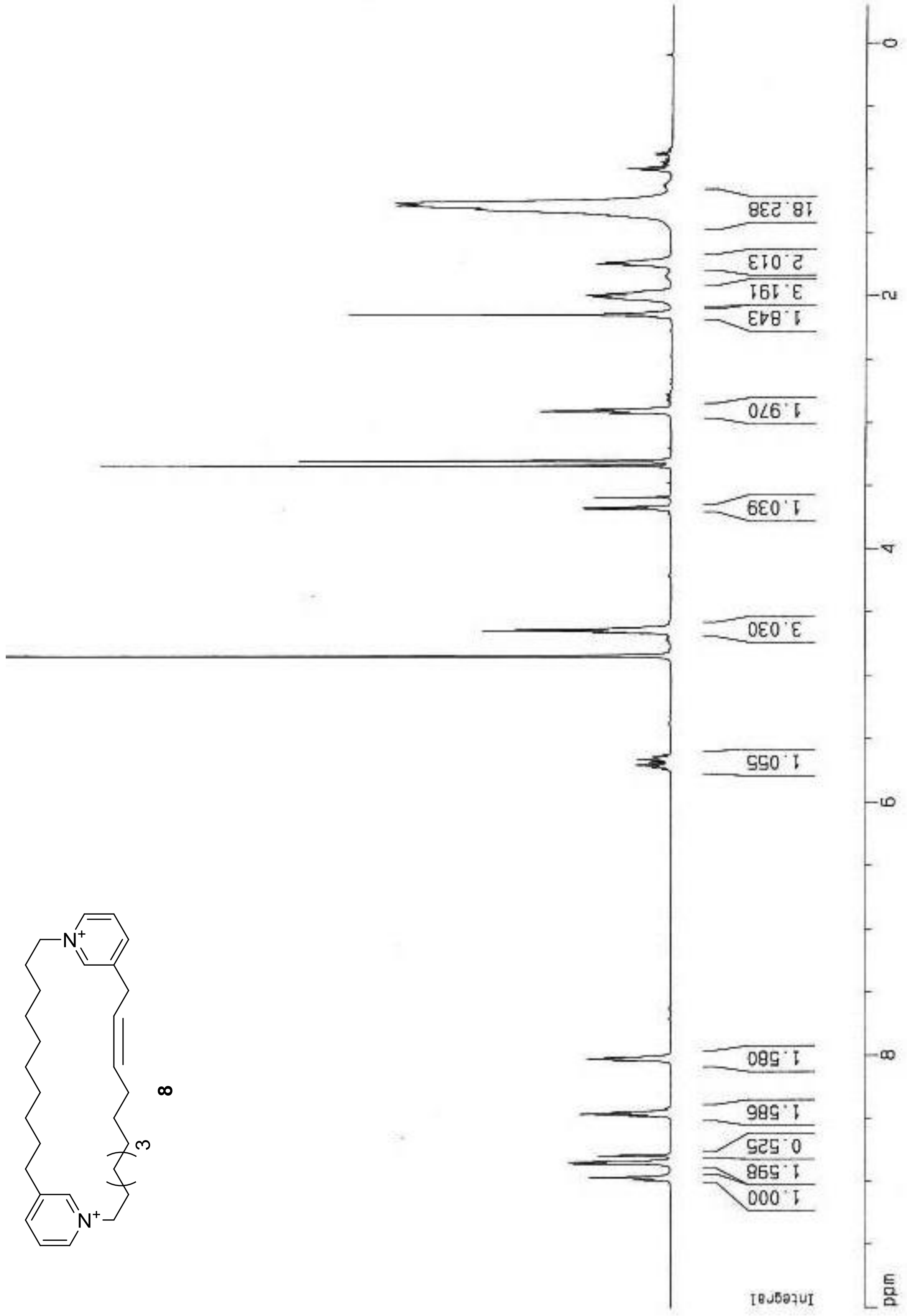

Figure S12. The 13C NMR (125 MHz, MeOH-d_4) spectrum of Compound **8**.

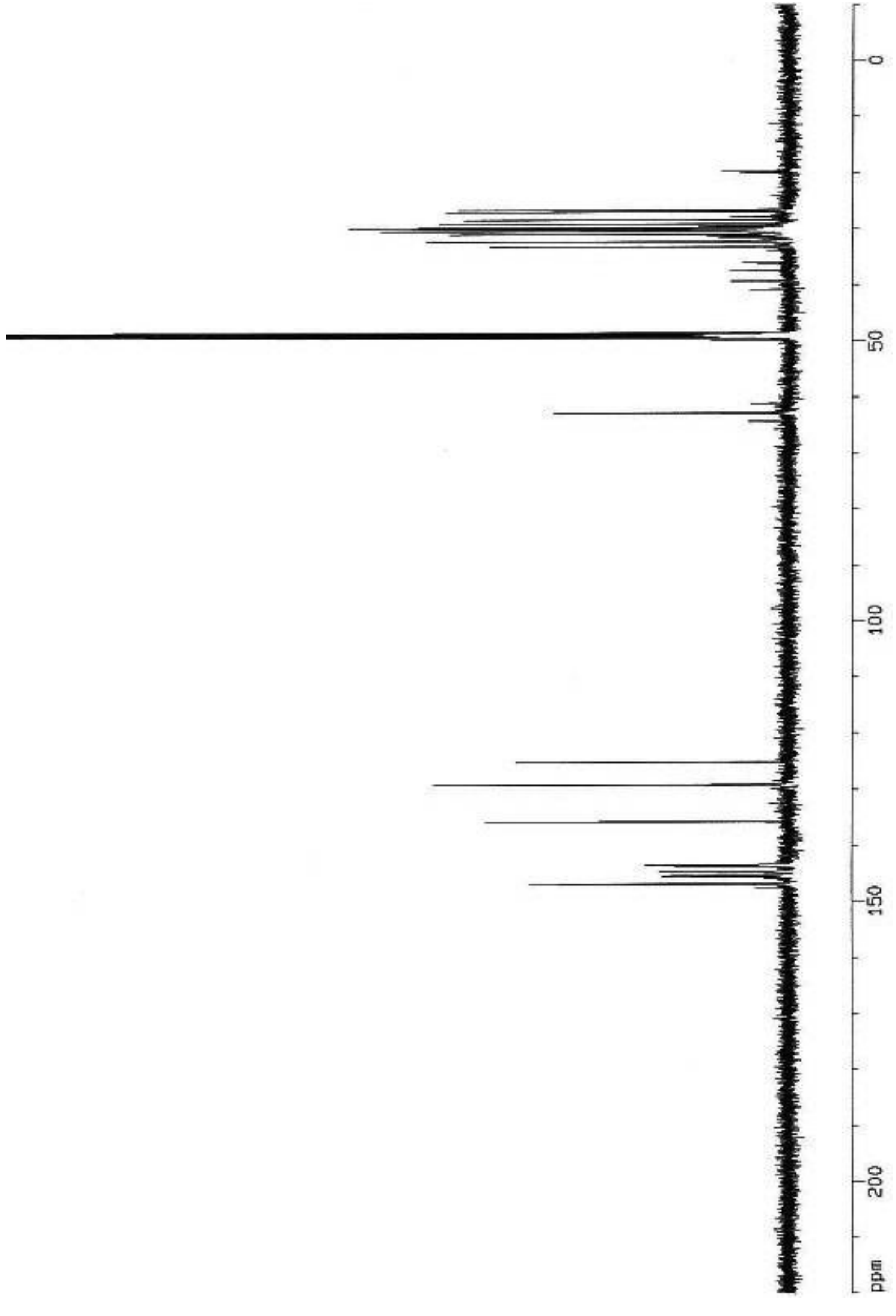

Figure S13. The 1H NMR (500 MHz, MeOH-d_4) spectrum of Compound **9**.

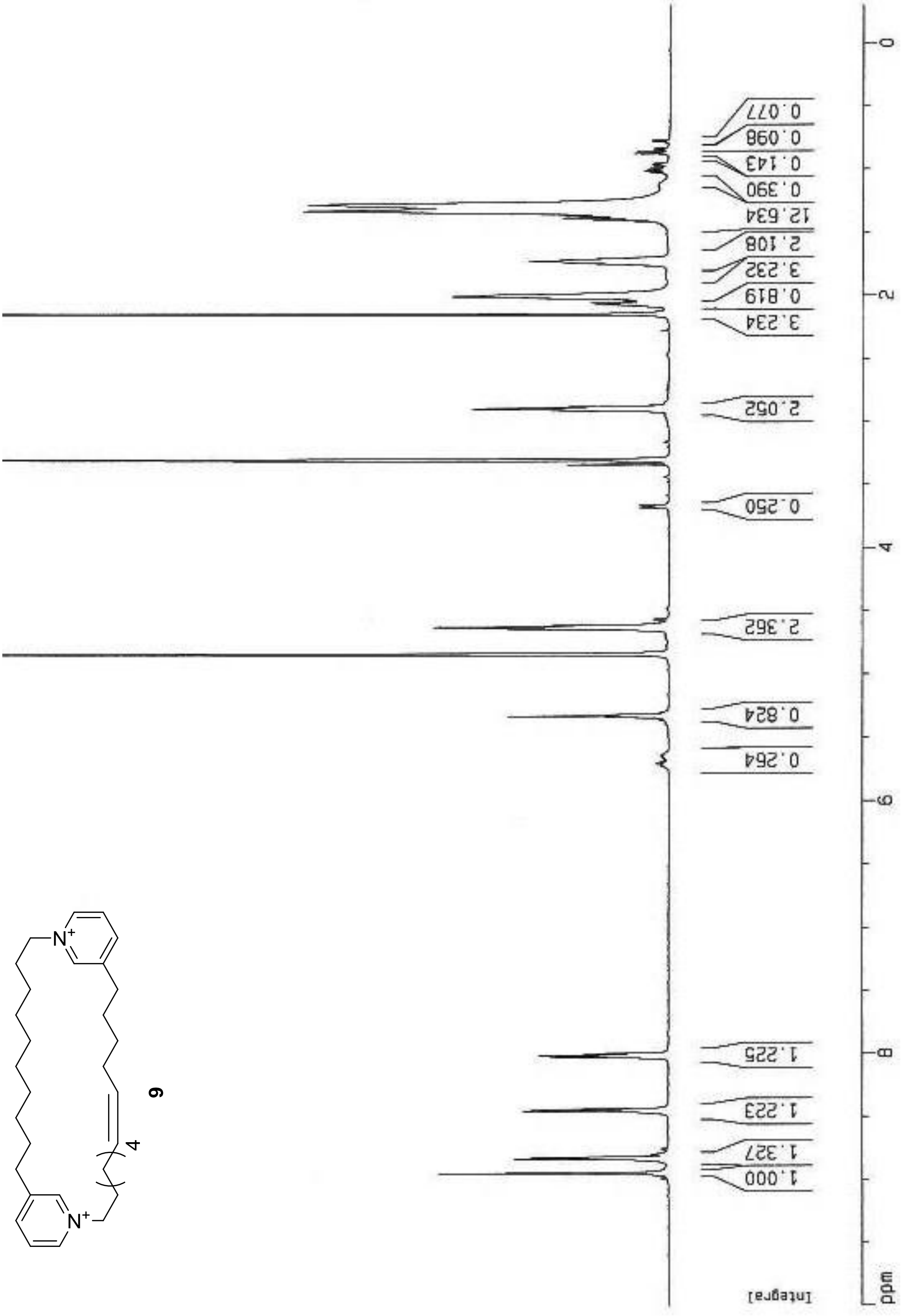

Figure S14. The 13C NMR (125 MHz, MeOH-d_4) spectrum of Compound **9**.

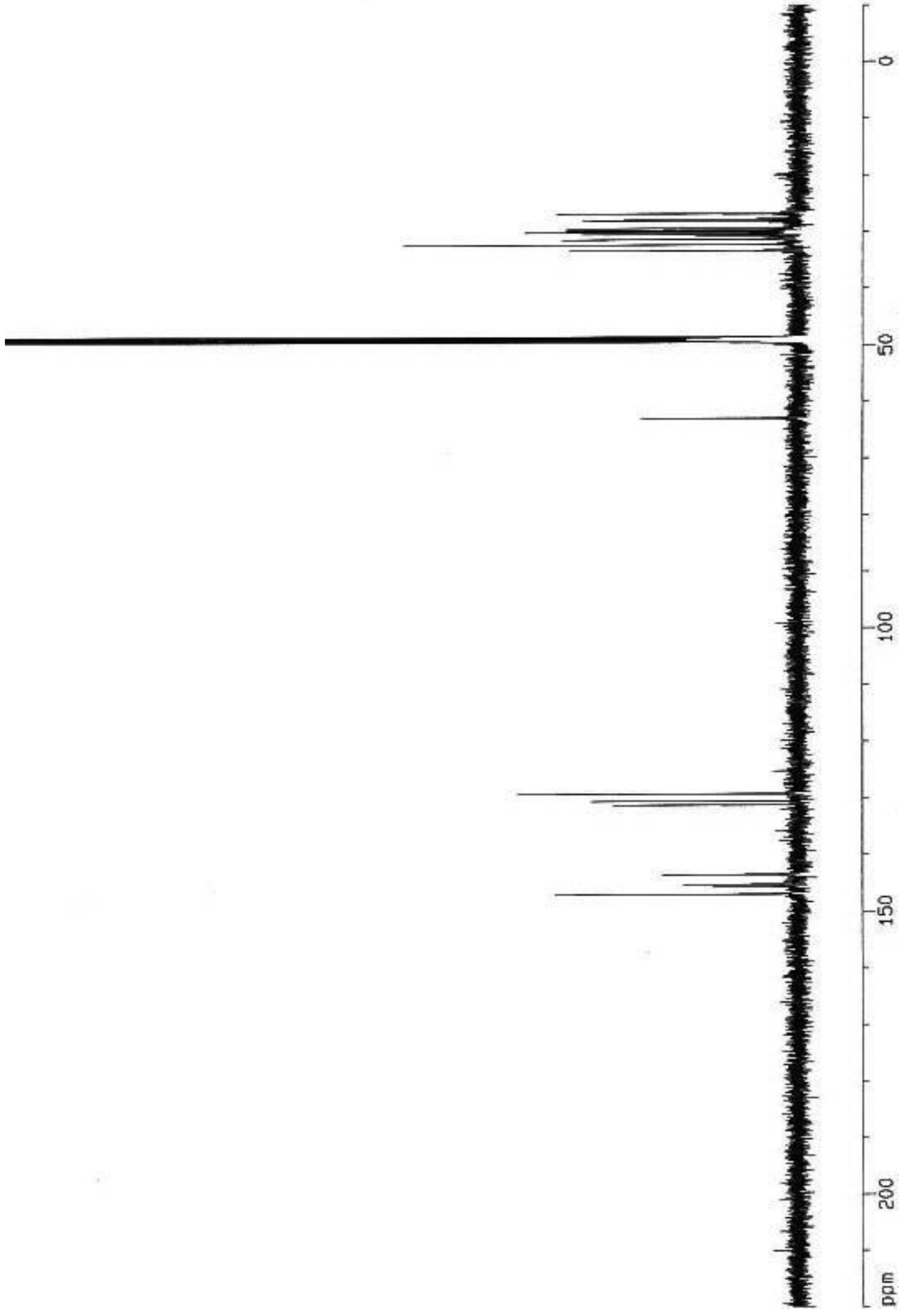

Figure S15. The 1H NMR (500 MHz, MeOH-d_4) spectrum of Compound **10**.

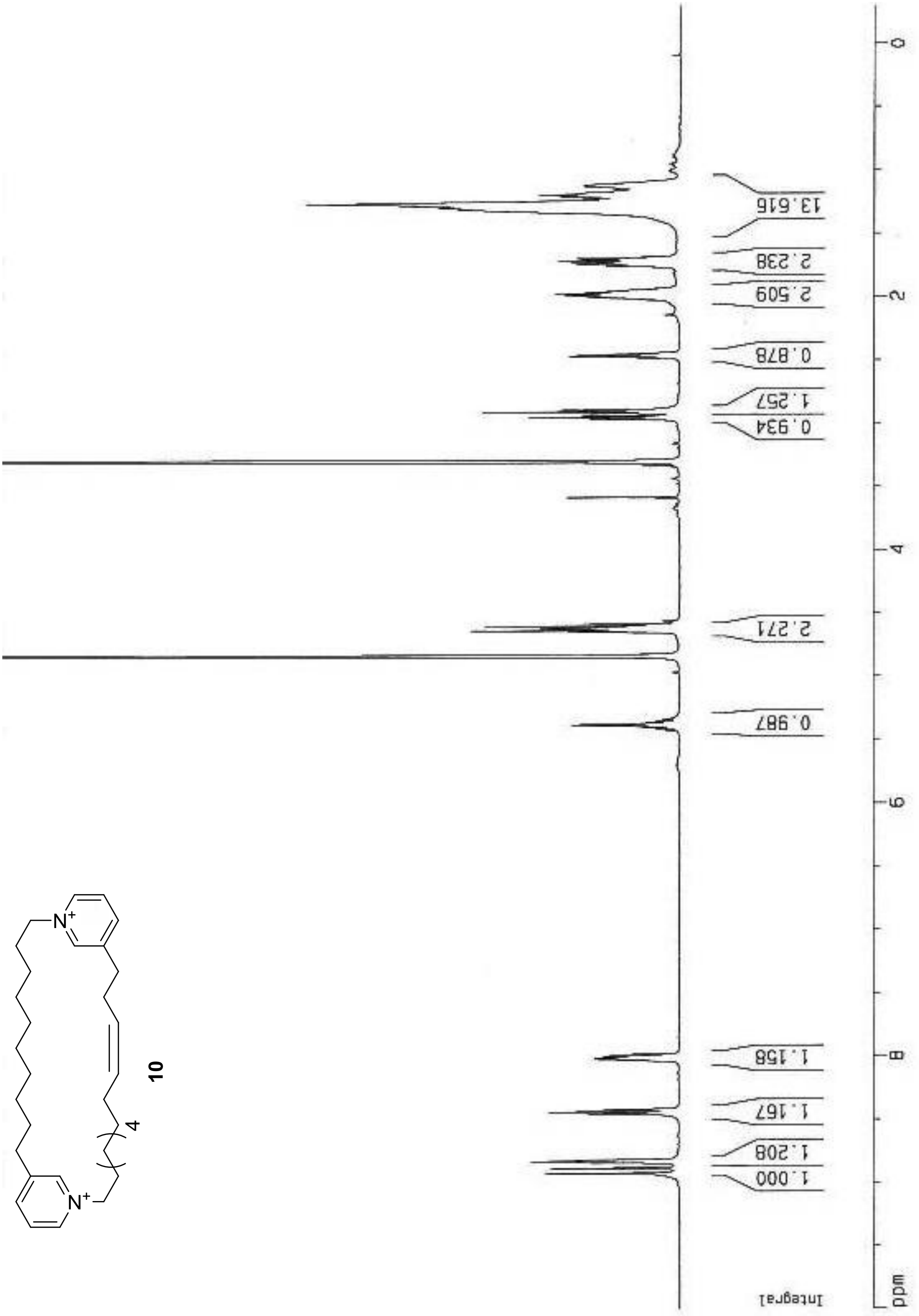

Figure S16. The 13C NMR (125 MHz, MeOH-d_4) spectrum of Compound **10**.

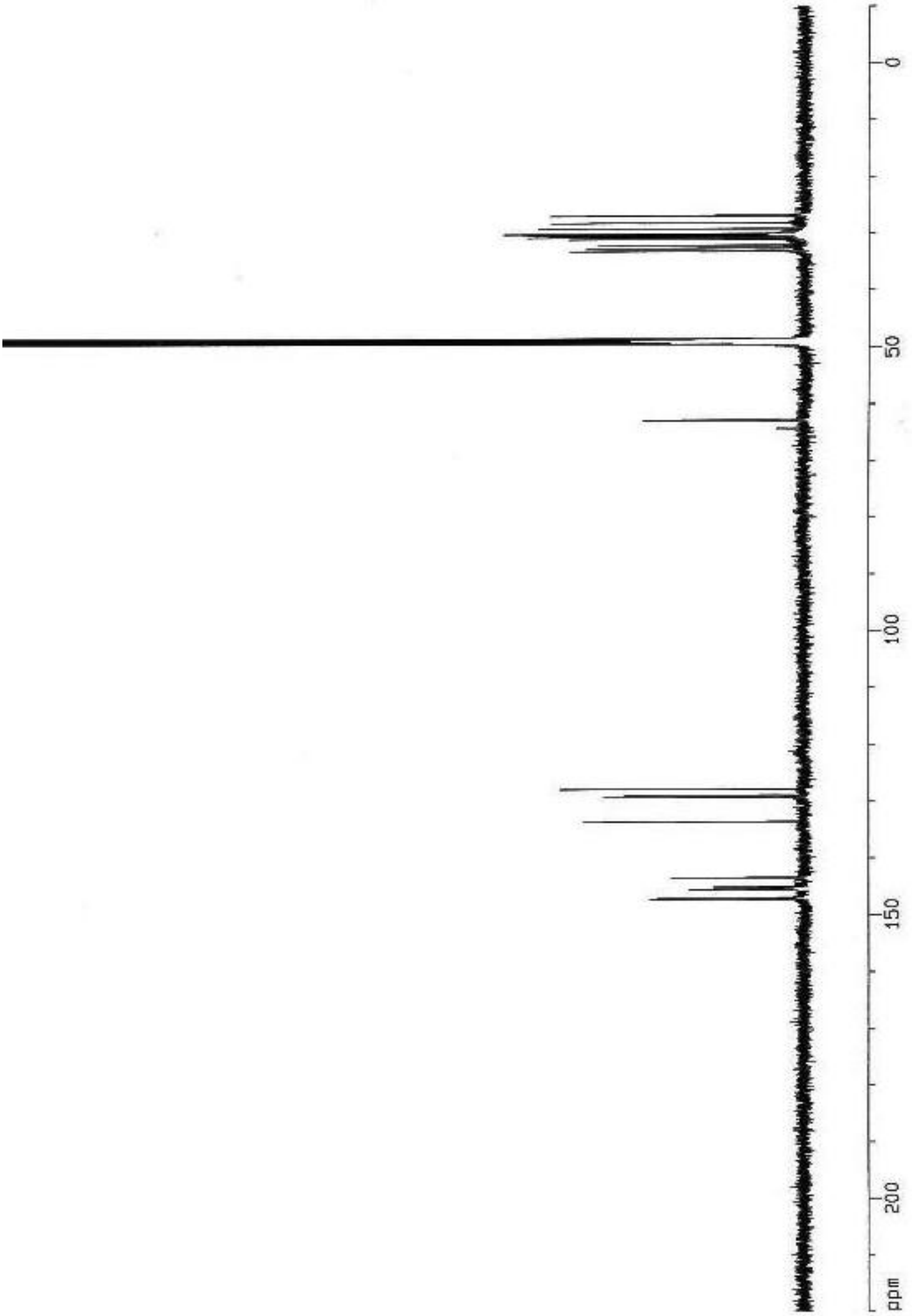

Figure S17. The cytotoxicity test results.

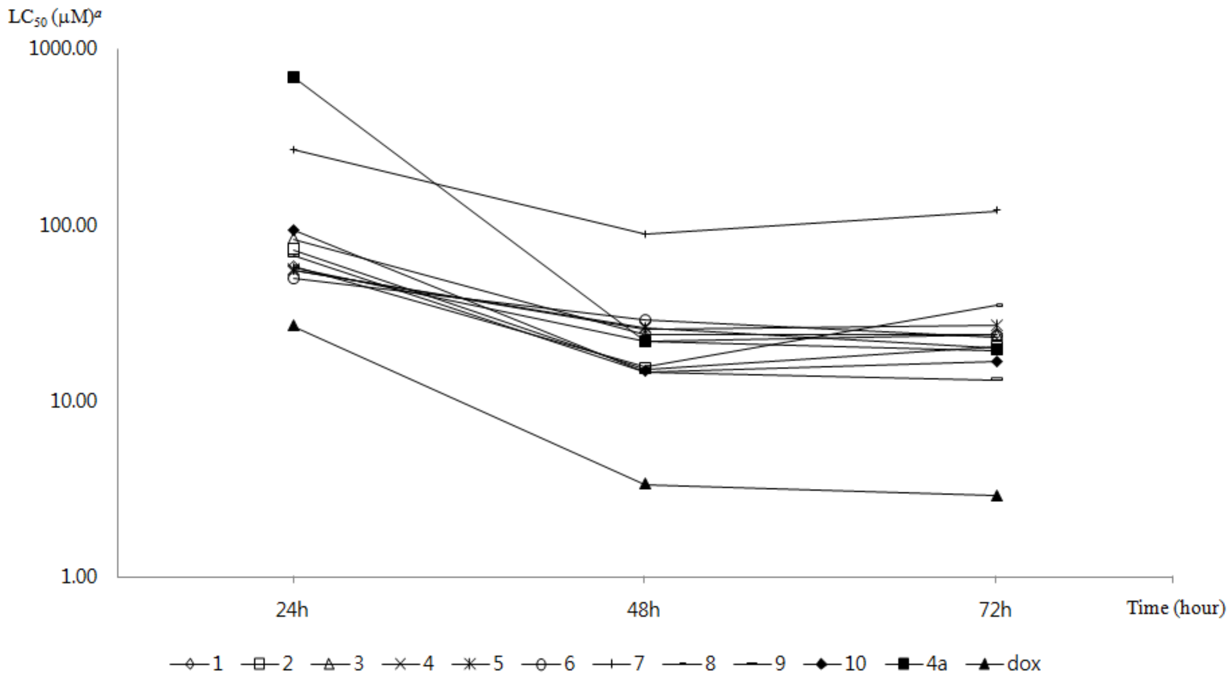

[a] Y axis is presented as log scale.

compd \ time	LC50 (μm)		
	24h	48h	72h
**1**	57.82	22.10	24.15
**2**	73.00	15.30	20.42
**3**	83.60	24.00	24.00
**4**	55.37	26.30	19.95
**5**	55.99	25.90	27.00
**6**	49.98	28.90	23.25
**7**	267.72	89.40	121.00
**8**	58.17	15.70	35.31
**9**	67.21	14.70	13.31
**10**	94.26	14.80	16.80
**4a**	696.35	21.90	19.47
dox	26.85	3.37	2.91
